# Ethylene increases the NaHCO_3_ stress tolerance of grapevines partially via the VvERF1B‐VvMYC2‐VvPMA10 pathway

**DOI:** 10.1111/pbi.14565

**Published:** 2025-01-07

**Authors:** Guangqing Xiang, Zongbao Fan, Shuxia Lan, Dezheng Wei, Yazhe Gao, Hui Kang, Yuxin Yao

**Affiliations:** ^1^ Key Laboratory of Biology and Genetic Improvement of Horticultural Crops in Huang‐Huai Region, Ministry of Agriculture College of Horticulture Science and Engineering, Shandong Agricultural University Tai‐An China; ^2^ National Center of Technology Innovation for Comprehensive Utilization of Saline‐Alkali Land Dongying China

**Keywords:** ethylene, NaHCO_3_ tolerance, VvERF1B, VvMYC2, VvPMA10

## Abstract

Here, we evaluated the role of ethylene in regulating the NaHCO_3_ stress tolerance of grapevines and clarified the mechanism by which *VvERF1B* regulates the response to NaHCO_3_ stress. The exogenous application of ACC and *VvACS3* overexpression in grapevines and grape calli revealed that ethylene increased NaHCO_3_ stress tolerance, and this was accompanied by increased plasma membrane H^+^‐ATPase (PMA) activity. The expression of *VvERF1B* was strongly induced by ACC, and overexpression of this gene in grapevines conferred increased NaHCO_3_ stress tolerance and enhanced PMA activity and H^+^ and oxalate secretion. Additionally, the function of *VvERF1B* was also verified using mutant transgenic grape calli and overexpression in *Arabidopsis* plants. The expression of *VvPMA10* was strongly induced following the overexpression of *VvERF1B* in grapevine roots, and *VvPMA10* was shown to regulate PMA activity, oxalate and H^+^ secretion, and NaHCO_3_ stress tolerance via its overexpression and mutation in grapevine roots, calli, and/or *Arabidopsis*. However, *VvPMA10* was not a direct target gene of VvERF1B but was directly transactivated by VvMYC2. The function of *VvMYC2* was shown to be similar to that of *VvPMA10* via its overexpression and mutation in grape calli. Additional experiments revealed that the interaction of VvERF1B with VvMYC2 increased its ability to activate *VvPMA10* expression and that VvMYC2 played a role in the VvERF1B‐mediated pathway. Overall, the VvERF1B‐VvMYC2‐VvPMA pathway played a role in regulating ethylene‐induced NaHCO_3_ stress tolerance in grapevines, and this process contributed to increases in PMA activity and H^+^ and oxalate secretion.

## Introduction

Soil alkalinity, which is primarily induced by sodium carbonate (Na_2_CO_3_) and sodium bicarbonate (NaHCO_3_), is one of the main factors limiting agricultural production across ~ 600 million acres of land worldwide (Zhang *et al*., 2023a). Alkaline salts induce osmotic stress, ion toxicity, high pH injury, and nutrient deficiencies in plants (Xu *et al*., 2013), which lead to more severe injuries compared with neutral salts. A recent study confirms that the weak acid ions from alkaline salts are the main factor contributing to alkaline stress (Chen *et al*., 2021). Although many studies have examined the mechanism underlying neutral salt stress tolerance, more studies are needed to clarify the mechanism underlying alkaline salt stress tolerance.

Ethylene is a key signalling molecule that mediates responses to various types of abiotic stress, including saline–alkali stress (Wang *et al*., 2023; Xu *et al*., 2019). Ethylene‐responsive factors (ERFs) are a large subfamily in the plant AP2/ERF superfamily, and ethylene‐related ERFs are important regulatory proteins in the ethylene signalling pathway. ERFs have been shown to regulate the tolerance of plants to various types of abiotic stress, including alkaline stress (Wu *et al*., 2022; Yu *et al*., 2017). A few studies have indicated that the overexpression of *MdERF1B* increases the cold tolerance of apple and *Arabidopsis* seedlings (Wang *et al*., 2021) and promotes anthocyanin and proanthocyanidin biosynthesis in apple calli (Zhang *et al*., 2018). *ERF1B* also plays a role in the reallocation of nitrate to the leaves and roots of *Arabidopsis* plants (Wang *et al*., 2022). Therefore, *ERF1B* might play a role in regulating multiple biological processes. Our previous study has found that the expression of *VvERF1B* is strongly induced by NaHCO_3_ stress (Xiang *et al*., 2019); however, the role of *VvERF1B* in regulating alkaline stress tolerance remains unclear.

MYC2, which is a member of the basic helix loop helix (bHLH) family, is the main downstream effector in the jasmonic acid (JA) signalling pathway (Lian *et al*., 2017). MYC2 might play a role in the ethylene signalling pathway. For example, MYC2 interacts with EIN3 and EIL1, two key transcription factors involved in ethylene signalling, and represses the expression of defence‐responsive genes (Song *et al*., 2014). Several studies have shown that MYC2 plays a role in regulating biotic and abiotic stress responses, growth and development, and the synthesis of specialized metabolites in plants (Luo *et al*., 2023). *MdMYC2* overexpression has been shown to increase the cold tolerance of apple calli by activating the expression of *MdCBF1* (Wang *et al*., 2019). OsMYC2‐like enhances salt tolerance by binding to the *OsCYP2* promoter in rice (Liu *et al*., 2022). MYC2 acts as a transcriptional activator in the ABA signalling pathway under drought stress (Wei *et al*., 2020). However, the role of MYC2 in regulating the response to alkaline stress has not yet been clarified.

Plasma membrane H^+^‐ATPase (PMA) is responsible for generating an electrochemical proton gradient by mediating the transport of H^+^ to the apoplast, which mediates the secondary transport of various chemical compounds (Michalak *et al*., 2022). PMA plays a key role in maintaining ion homeostasis and intracellular pH and increasing tolerance to various types of abiotic stress (Michalak *et al*., 2022). In *Arabidopsis*, the PMAs AHA2 and AHA7 have been shown to mediate H^+^ efflux in the root elongation zone and root hair zone, respectively (Yuan *et al*., 2017), and AHA2 activity is closely correlated with alkaline stress tolerance (Yang *et al*., 2019a). PMA has also been shown to contribute to the secretion of organic acids into the rhizosphere (Crombez *et al*., 2019). The accumulation and secretion of organic acids help maintain pH and ion homeostasis of the roots and rhizosphere, which prevents injuries caused by alkaline salts (Jia *et al*., 2019; Yang, 2012). The mechanisms regulating organic acid biosynthesis and secretion vary among plants (Alhendawi *et al*., 1997; Guo *et al*., 2010; Yang *et al*., 2007). Grape roots mainly accumulate and secrete oxalic acid under NaHCO_3_ stress (Xiang *et al*., 2019). By contrast, *Puccinellia tenuiflora* and maize roots primarily accumulate and secrete citric acid and malic acid, respectively, under alkali stress (Alhendawi *et al*., 1997; Guo *et al*., 2010). Previous studies have indicated that PMAs are involved in responses to salt and alkaline stress (Yang *et al*., 2019a); however, how plants sense these stresses and alter PMA activity in response to these stresses remains unclear.

Grapevines are an economically important fruit that are frequently exposed to alkaline and salt stress. In China, most arable land is used for the cultivation of grain crops; grapevines and other fruit trees are thus often cultivated on marginal land with saline–alkali soil. The development and the use of saline–alkali land have been a major focus of Chinese policymakers. There is thus a need to clarify the mechanism underlying the tolerance of grapevines to saline–alkali stress, as this will aid future molecular breeding efforts. The aim of this study was to investigate the effects of ethylene on grapevine NaHCO_3_ stress tolerance and clarify the role of the VvERF1B‐VvMYC2‐VvPMA10 pathway in increasing the NaHCO_3_ stress tolerance of grapevines. We also evaluated the efficacy of using *VvERF1B* to genetically engineer NaHCO_3_‐tolerant grapevines by up‐regulating the above pathway.

## Results

### Ethylene enhances the NaHCO_3_ stress tolerance of grapevines and increases PMA activity in grapevine roots and calli under NaHCO_3_ stress

**Figure 1:**
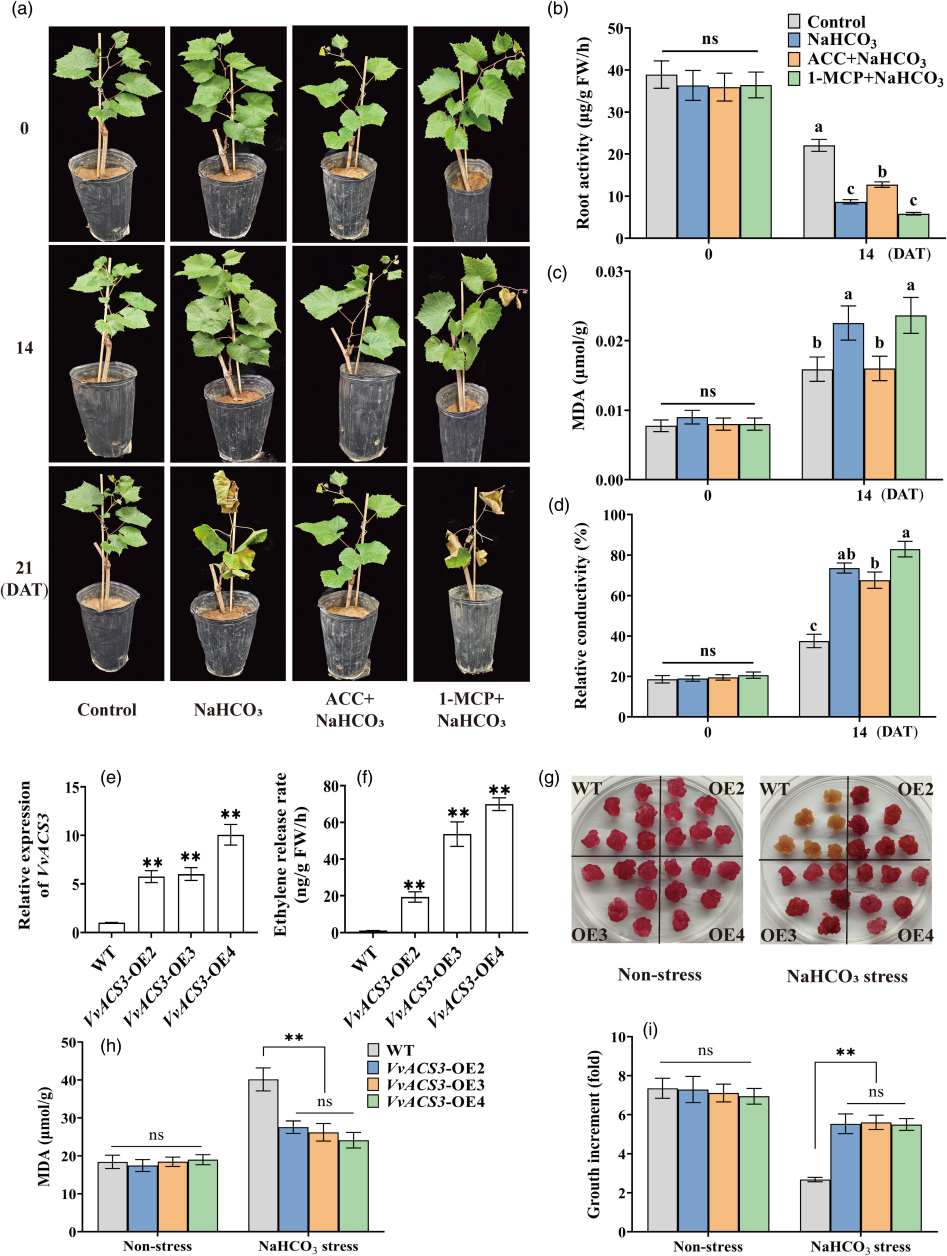
Effects of the exogenous application of ACC and 1‐MCP and *VvACS3* overexpression on the NaHCO_3_ stress tolerance of grapevines and calli. (a) Phenotypes of grapevines grown in a greenhouse under natural conditions and under various treatments (0, 14, and 21 DAT). The vines were treated with 100 mm NaHCO_3_ and 50 μm ACC for 3 h, followed by 100 mm NaHCO_3_ (ACC + NaHCO_3_) and 0.2 μm 1‐MCP for 3 h, and 100 mm NaHCO_3_ (1‐MCP + NaHCO_3_). (b–d) Root activity, MDA content, and relative conductivity of grapevines under various treatments at 0 and 14 DAT. (e, f) Expression and ethylene release rate in *VvACS3*‐overexpressing calli. (g–i) Phenotypes, MDA content, and growth increase of *VvACS3*‐overexpressing calli at 21 days after 2.5 mm NaHCO_3_ treatment. DAT, days after treatment. Data represent means ± standard deviation (SD) of three biological experiments. Significant differences between WT and transgenic lines were calculated using Student's *t*‐test. ** *P*‐value <0.01, and different lowercase letters represent significant differences at *P* < 0.05.

The results of our previous study showed that NaHCO_3_ stress significantly induces *VvACS3* transcription and ethylene release in grape roots (Xiang *et al*., 2019). To clarify the role of ethylene in regulating NaHCO_3_ stress tolerance, grape cuttings were treated with ACC (the precursor of ethylene synthesis) and 1‐MCP (the inhibitor of ethylene signalling) for 3 weeks. NaHCO_3_ stress resulted in leaf withering and defoliation at 21 days after treatment (DAT). By contrast, ACC application alleviated NaHCO_3_ injury, and 1‐MCP application resulted in a more severe phenotype (Figure 1a). ACC application also significantly increased root activity and reduced the malondialdehyde (MDA) content and relative conductivity; 1‐MCP application had the opposite effect (Figure 1b–d). In addition, three lines of *VvACS3*‐overexpressing grape calli were obtained to evaluate the effect of endogenous ethylene on NaHCO_3_ stress tolerance. The transgenic lines had a higher ethylene release rate compared with wild‐type (WT) calli (Figure 1f). The phenotypes of transgenic and non‐transgenic calli were similar under normal conditions (Figure 1g). Under NaHCO_3_ treatment, WT calli became brown (Figure 1g); the MDA content was lower in transgenic calli than in WT calli, and transgenic calli grew more rapidly than WT calli (Figure 1h,i). Collectively, ethylene positively contributed to the enhanced NaHCO_3_ stress tolerance of grapevines and grape calli.

**Figure 2:**
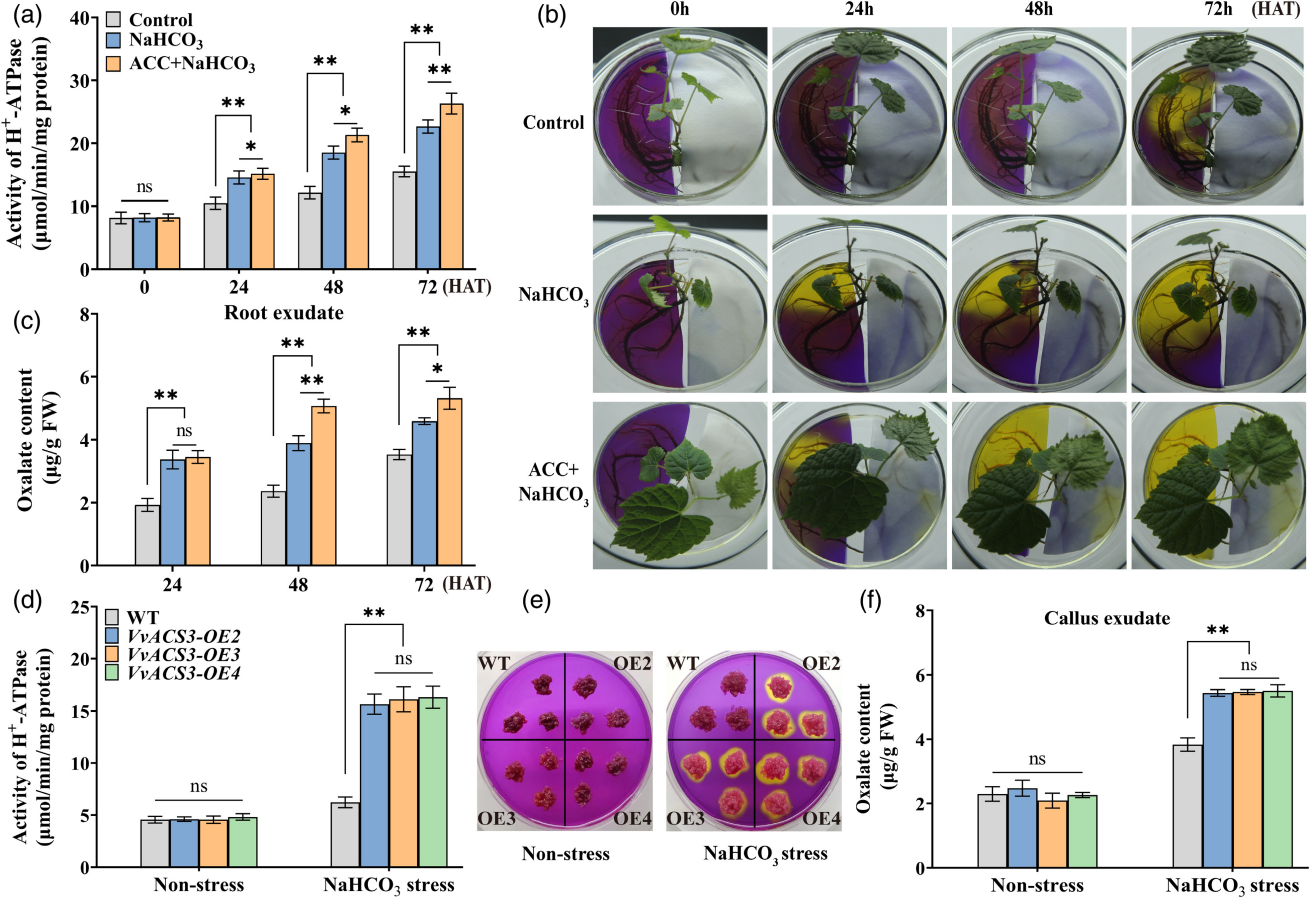
Changes in PMA activity and H^+^ and oxalate secretion in roots and calli under NaHCO_3_ stress. (a) PMA activity in roots under NaHCO_3_ treatment. (b) Rhizosphere acidification is indicated by pH‐sensitive bromocresol purple. Vine roots were divided into two equal parts; one part was placed in a medium containing 100 mm NaHCO_3_, and another part was placed in a medium containing 0.006% bromocresol purple. For NaHCO_3_ + ACC treatment, the roots were pretreated with 50 μm ACC for 3 h before being covered by the medium. (c) Oxalate content in root exudates. (d–f) PMA activity and H^+^ and oxalate secretion in *VvACS3*‐overexpressing calli at 2 days after subculture in the medium; calli were treated with 100 mm NaHCO_3_ for 6 h before subculture. Values represent the means ± SD of three replicates. * Significant difference, *P* < 0.05; ** highly significant difference, *P* < 0.01.

We also determined the effect of ACC on PMA activity. The PMA activity of the roots was significantly higher in the NaHCO_3_ and NaHCO_3_ + ACC treatments than in the control (Figure 2a). PMAs are the major pump responsible for root H^+^ efflux (Fuglsang *et al*., 2007); therefore, we measured the H^+^ secretion level. Large areas of yellow that were observed in the NaHCO_3_ + ACC treatment were not observed in the NaHCO_3_ treatment at the same time points (Figure 2b), indicating that large amounts of H^+^ were secreted when ACC was applied. H^+^ could combine organic acid anions to form organic acid, and the results of our previous study have shown that oxalate is the main organic acid produced under NaHCO_3_ stress (Xiang *et al*., 2019). We thus measured the oxalate secretion level; the oxalate content was higher in root exudates in the NaHCO_3_ + ACC treatment than in the NaHCO_3_ treatment at 24, 48, and/or 72 h after treatment (HAT) (Figure 2c). Additionally, *VvACS3* overexpression significantly promoted PMA activity, H^+^ secretion (indicated by the yellow colour), and oxalate secretion in grape roots under NaHCO_3_ treatment (Figure 2d–f). *VvACS3* overexpression did not significantly affect the above parameters under non‐stress conditions. Overall, our findings indicated that ethylene increased PMA activity, which might promote H^+^ and oxalate secretion under NaHCO_3_ stress.

### *VvERF1B* enhances NaHCO_3_ stress tolerance and PMA activity in transgenic grapevines, calli, and *Arabidopsis* plants

**Figure 3:**
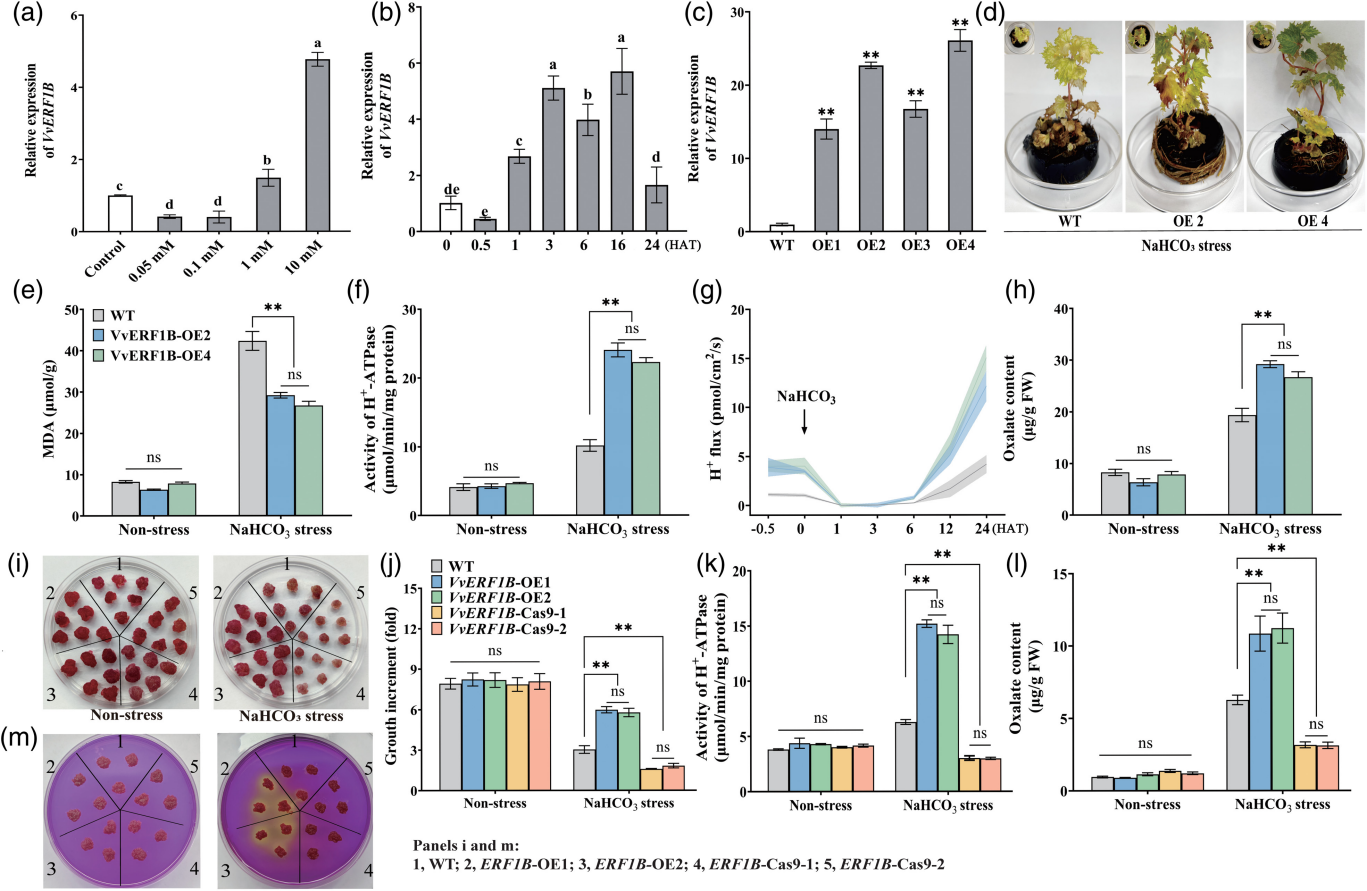
The role of *VvERF1B* in increasing NaHCO_3_ stress tolerance and modifying PMA activity, H^+^ efflux, and oxalate secretion in grapevines and calli. (a, b) The expression of *VvERF1B* in roots treated with different concentrations of ACC at 3 h (a) and with 10 mm ACC at different time points (b). (c) The expression of *VvERF1B* in the four transgenic grapevine lines. (d–h) Phenotypes of WT and *VvERF1B*‐overexpressing grapevines (d) and corresponding physiological parameters, including the MDA content (e), PMA activity (f), H^+^ efflux rate, and oxalate content in their roots (h) under 100 mm NaHCO_3_ treatment for 5 days. (i–m) Phenotype evaluation (i), growth increase (j), PMA activity (k), and levels of oxalate (l) and H^+^ (m) secretion in WT, *VvERF1B*‐overexpressing, and mutant grape calli. The calli in panels i–l were treated with 2.5 mm NaHCO_3_ for 21 days; the calli in panel m were treated with 100 mm NaHCO_3_ for 6 h and then were subcultured on medium containing 0.006% bromocresol purple. ns, not significant. Values are the means of three replicates, and error bars denote the SD. ** highly significant difference, *P* < 0.01.

The expression of *VvERF1B* was strongly induced by NaHCO_3_ stress according to the RNA sequencing data (Xiang *et al*., 2019). We found that the expression of *VvERF1B* was significantly induced by 10 mm ACC (Figure 3a,b). To clarify the role of *VvERF1B* in regulating NaHCO_3_ stress tolerance, we generated *VvERF1B*‐overexpressing grapevines. We performed assays on lines 2 (OE2) and 4 (OE4), which had high *VvERF1B* expression (Figure 3c). Following exposure to 100 mm NaHCO_3_ stress for 5 days, the phenotypes of transgenic grape shoot cultures were less severe than those of WT plants (Figure 3d). *VvERF1B* overexpression decreased the MDA content and increased PMA activity, the H^+^ efflux rate, and oxalate secretion (Figure 3e–h).

To further clarify the function of *VvERF1B*, we obtained two lines of *VvERF1B*‐overexpressing and mutant transgenic grape calli (Figure S1). Under NaHCO_3_ stress, *VvERF1B* overexpression and mutation increased and reduced the growth of transgenic calli, respectively (Figure 3i). Similar changes in PMA activity, H^+^ secretion, and oxalate secretion were observed (Figure 3j–m). Overexpression of *VvERF1B* in *Arabidopsis* plants revealed that *VvERF1B* plays a role in increasing NaHCO_3_ stress tolerance (Figure S2).

Overall, *VvERF1B* overexpression enhanced NaHCO_3_ stress tolerance and PMA activity, and this was accompanied by increased H^+^ efflux and oxalate secretion; mutation of *VvERF1B* had the opposite effect.

### VvERF1B indirectly regulates the expression of *VvPMA10* , which increases NaHCO_3_ stress tolerance in grape roots, calli, and *Arabidopsis* plants

**Figure 4:**
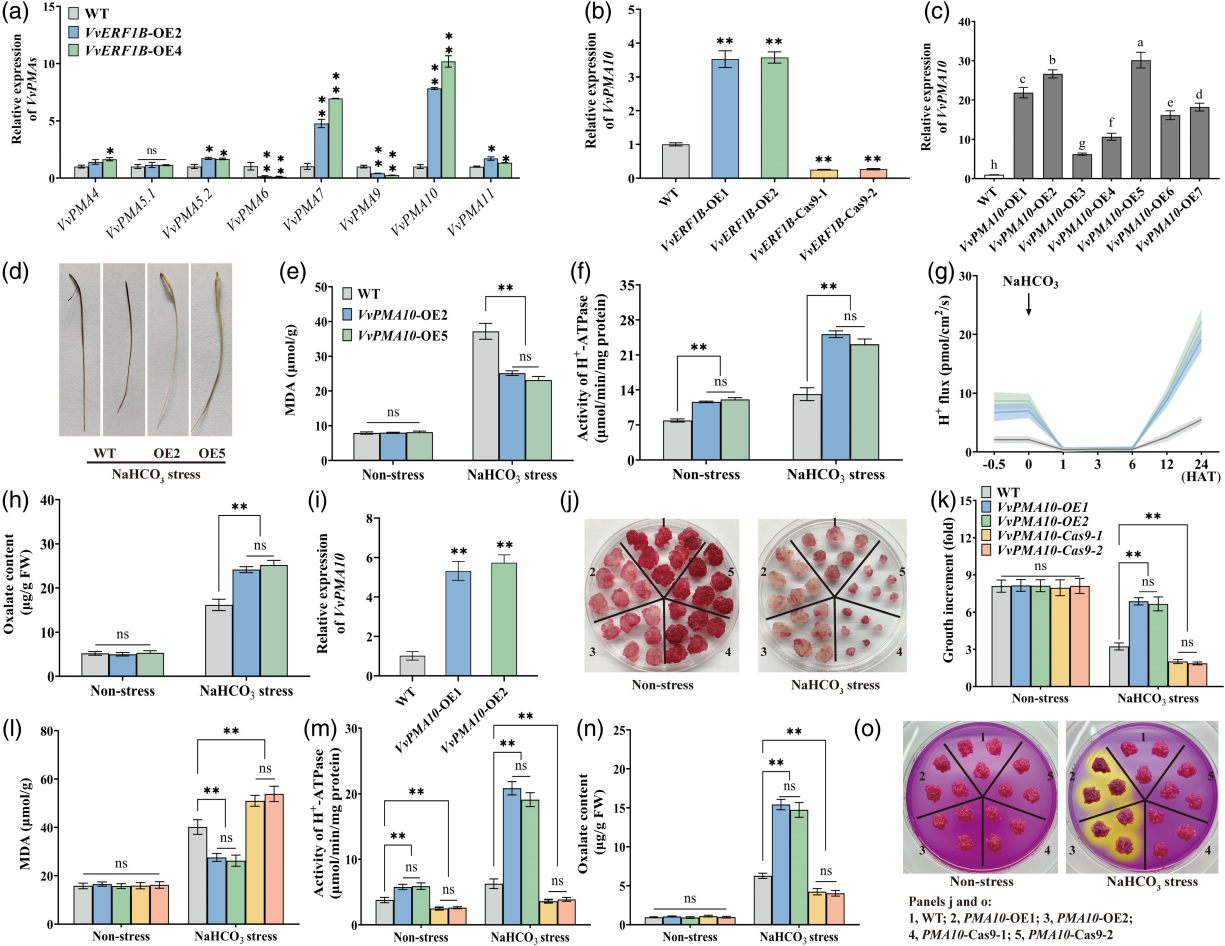
The role of *VvPMA10* in increasing NaHCO_3_ stress tolerance and modifying PMA activity in grapevines and calli. (a) Relative expression of the eight PMA genes in *VvERF1B*‐overexpressing grapevines. (b) Expression of *VvPMA10* in *VvERF1B*‐overexpressing and mutant grape calli. (c) Identification of *VvPMA10*‐overexpressing grape roots via *Agrobacterium rhizogenes*‐mediated gene transformation using qRT‐PCR. (d–h) Phenotypes of WT and transgenic roots and corresponding physiological parameters, including the MDA content (e), PMA activity (f), H^+^ efflux rate (g), and oxalate secretion (h) under 50 mm NaHCO_3_ treatment for 3 days. (i) Expression of *VvPMA10* in WT and *VvPMA10*‐overexpressing grape calli. (j–o) Phenotype (j), growth increase (k), MDA content (l), PMA activity (m), and oxalate (n) and H^+^ (o) secretion of WT, *VvPMA10*‐overexpressing, and mutant grape calli under 2.5 mm NaHCO_3_ for 21 days. The calli in panel m were treated with 100 mm NaHCO_3_ for 6 h and then were subcultured on medium containing 0.006% bromocresol purple. Values represent the means ± SD of three replicates. * Significant difference, *P* < 0.05; ** highly significant difference, *P* < 0.01.

Given that PMA activity was increased in the roots of *VvERF1B*‐overexpressing grapevines, the expression of eight PMA genes in *VvERF1B*‐overexpressing grapevines was determined. The expression of *VvPMA10* was strongly induced by *VvERF1B* overexpression in grapevine roots (Figure 4a). Similar results were observed in *VvERF1B*‐overexpressing grape calli. Mutation of *VvERF1B* resulted in the down‐regulation of the expression of *VvPMA10* (Figure 4b). Thus, *VvPMA10* might be a target gene of VvERF1B. Although two ERE elements are present in the *VvPMA10* promoter, electrophoretic mobility shift assay (EMSA) and yeast one‐hybrid (Y1H) experiments revealed that VvERF1B did not bind to the *VvPMA10* promoter (Figure S3).

The function of *VvPMA10* was evaluated by overexpressing it in grapevine roots. A total of seven lines were obtained, and lines 2 and 5 were used in subsequent experiments (Figure 4c). The transgenic roots had a less severe phenotype and a lower MDA content after NaHCO_3_ treatment compared with WT roots (Figure 4d,e). *VvPMA10* overexpression led to increases in PMA activity, the H^+^ efflux rate, and oxalate secretion (Figure 4f–h). *VvPMA10* overexpression (Figure 4i) increased the tolerance of grape calli to NaHCO_3_ (Figure 4j), as indicated by their greater growth (Figure 4k) and lower MDA content (Figure 4l) compared with WT plants. Simultaneously, PMA activity and oxalate and H^+^ secretion were enhanced in *VvPMA10*‐overexpressing calli (Figure 4m–o). By contrast, mutation of *VvPMA10* had the opposite effect (Figures S4; Figure 4m–o). *VvPMA10* overexpression increased the NaHCO_3_ stress tolerance of *Arabidopsis* (Figure S5).

Overall, *VvPMA10* was indirectly transactivated by VvERF1B, and its overexpression promoted PMA activity, oxalate and H^+^ secretion, and NaHCO_3_ stress tolerance.

### VvMYC2 transactivates *VvPMA10* expression and enhances the NaHCO_3_ stress tolerance of grape calli

**Figure 5:**
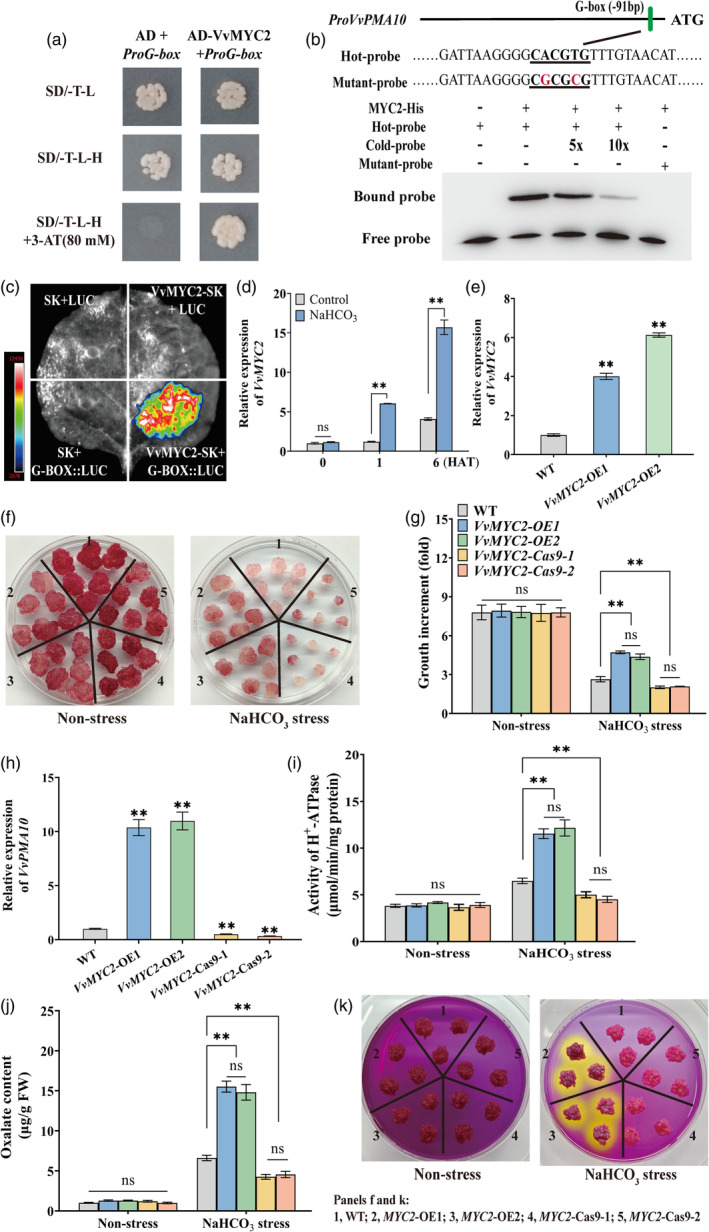
Characterization of the transcriptional activation of *VvPMA10* by VvMYC2 and the role of *VvMYC2* in increasing NaHCO_3_ stress tolerance of calli. (a) Y1H assays. AD+ProG‐box indicates empty pGADT7 + *ProG‐box*::pHIS2, and AD‐VvMYC2 + ProG‐box indicates VvMYC2‐pGADT7 + *ProG‐box*::pHIS2. (b) Interaction of the VvMYC2 fusion protein with the DNA probes for the G‐box and mutant G‐box within the *VvPMA10* promoter in an EMSA. (c) Representative images of tobacco leaves at 48 h after infiltration. (d) Expression of *VvMYC2* in grapevine roots under 100 mm NaHCO_3_. (e) Identification of *VvMYC2*‐overexpressing grape calli using qRT‐PCR. (f–k) Phenotypes (f), growth (g), expression of *VvPMA10* (h), PMA activity (i), and oxalate (j) and H^+^ secretion level (k) of WT, *VvMYC2*‐overexpressing, and mutant grape calli under 2.5 mm NaHCO_3_ for 21 days. The calli in panel k were treated with 100 mm NaHCO_3_ for 6 h and then were subcultured on a medium containing 0.006% bromocresol purple. Values represent the means ± SD of three replicates. ** highly significant difference, *P* < 0.01.

To clarify the molecular pathways underlying the *VvERF1B*‐induced expression of *VvPMA10*, yeast one‐hybrid (Y1H) screening was performed to identify upstream transcription factors of *VvPMA10*, and this revealed the candidate transcription factor *VvMYC2* (VIT_215s0048g02820). The potential binding elements of VvMYC2 (Two G‐box, four E‐box, and four MYC) were used to perform Y1H assays, and VvMYC2 was found to bind to the G‐box (CACGTG) (Figure 5a; Figure S6). EMSAs and luciferase (LUC) assays were performed to verify the results (Figure 5b,c).

The expression of *VvMYC2* was strongly induced by NaHCO_3_ treatment (Figure 5d). To verify the role of *VvMYC2* in regulating NaHCO_3_ stress tolerance, two lines of *VvMYC2*‐overexpressing and mutant grape calli were obtained (Figure 5e, Figure S7). *VvMYC2* overexpression and mutation increased and decreased growth increment of grape calli, respectively, compared with WT plants (Figure 5f,g), suggesting that *VvMYC2* plays a role in increasing NaHCO_3_ stress tolerance. *VvMYC2* overexpression up‐regulated *VvPMA10* expression (Figure 5h), and this resulted in increased PMA activity, as well as oxalate and H^+^ secretion (Figure 5i–k); by contrast, mutation of *VvMYC2* had the opposite effect (Figure 5h–k).

Overall, VvMYC2 increased NaHCO_3_ stress tolerance by transactivating its target gene *VvPMA10*.

### VvERF1B interacts with VvMYC2, which promotes the up‐regulation of *VvPMA10* expression and NaHCO_3_ stress resistance in grape calli

**Figure 6:**
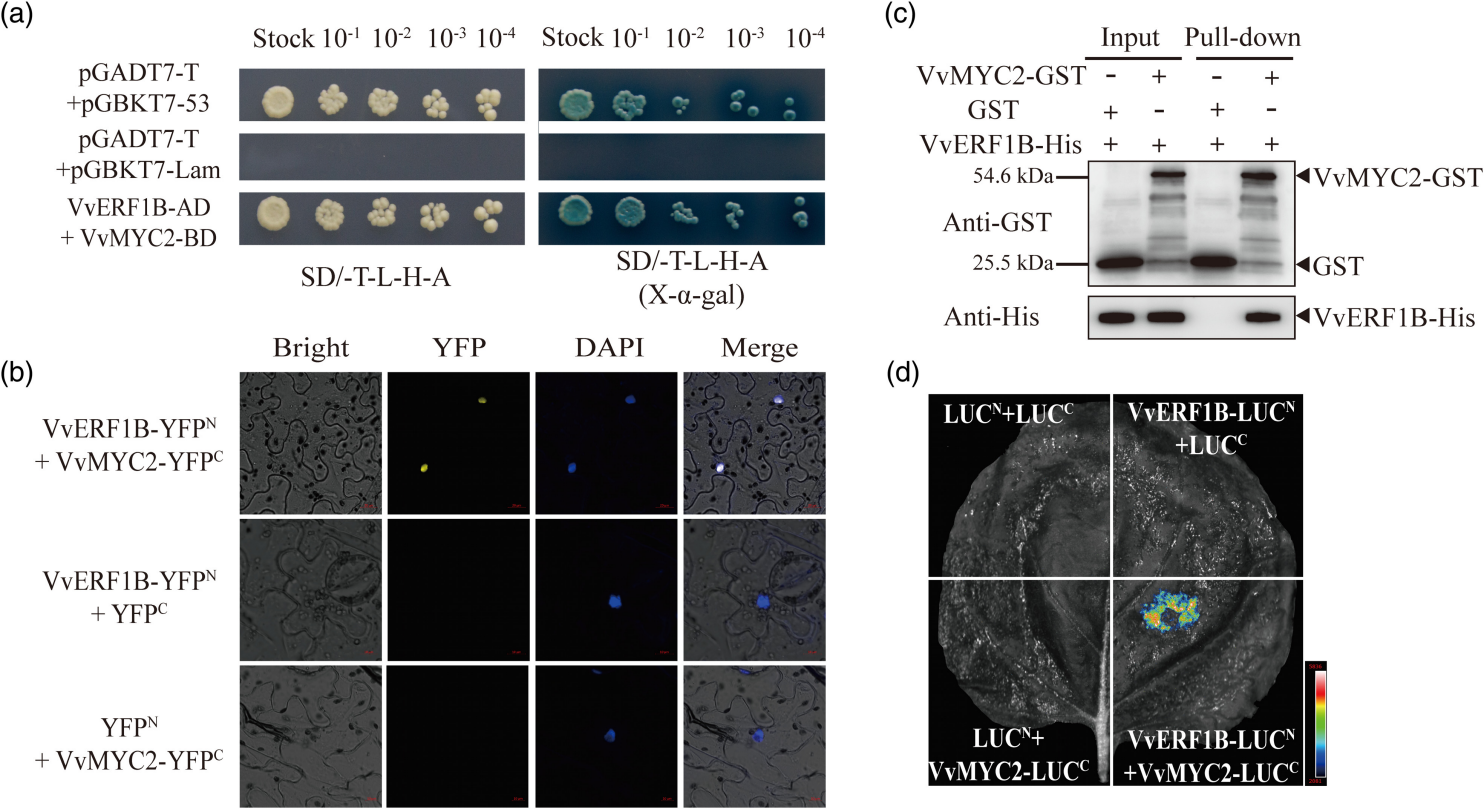
Interaction of VvERF1B with VvMYC2 *in vitro* and *in vivo*. (a) The VvERF1B‐pGADT7 interaction with VvMYC2‐pGBKT7 in a Y2H system. (b) Confirmation of the interaction of VvERF1B with VvMYC2 by BiFC in *N. benthamiana* leaf epidermal cells, as indicated by a yellow fluorescent signal. (c) VvERF1B interacted with VvMYC2 in pull‐down assays. “+” and “−” indicate the presence and absence of the indicated protein, respectively. (d) Luciferase complementarity assay showing that VvERF1B interacts with VvMYC2.

**Figure 7:**
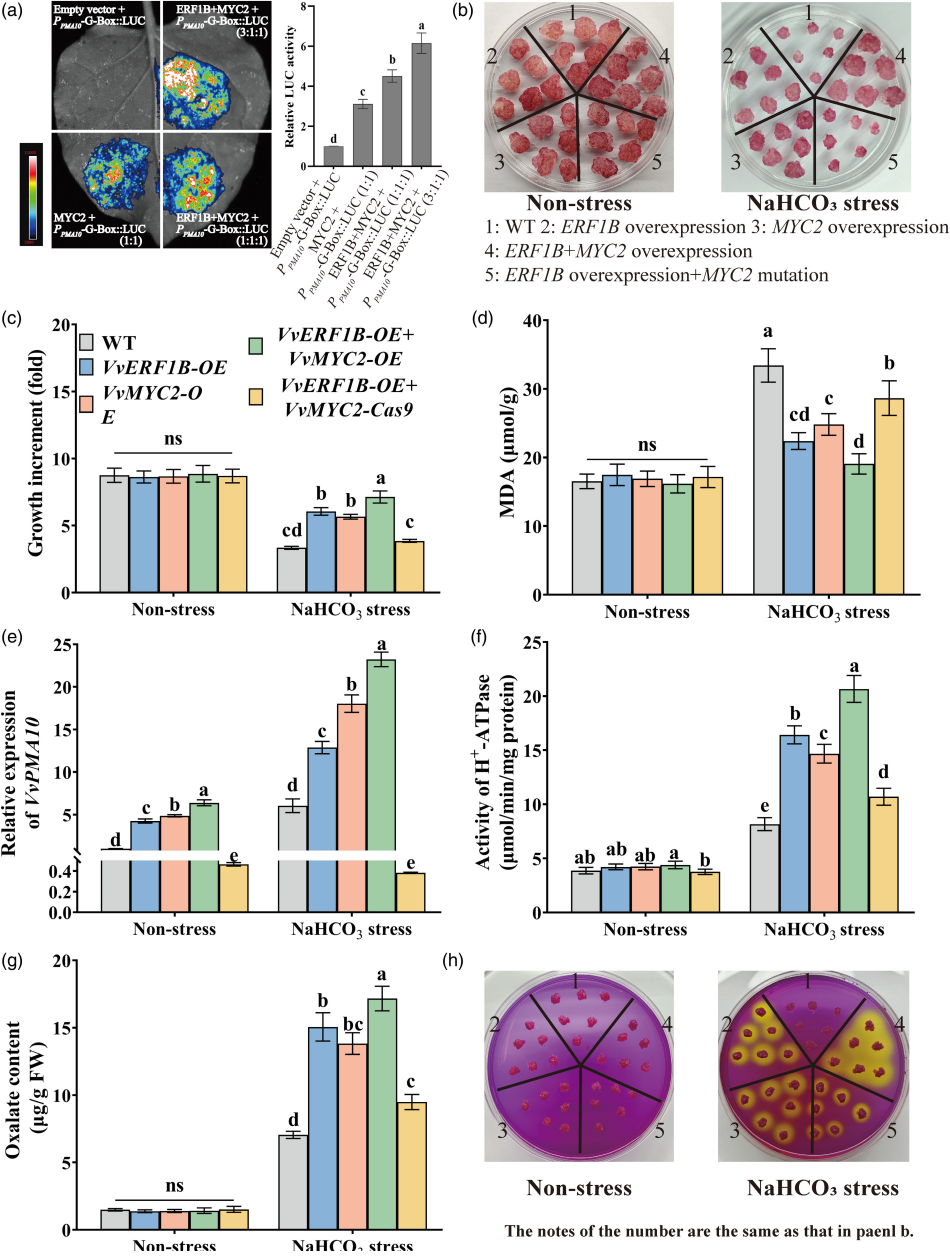
Effects of the VvERF1B–VvMYC2 complex on *VvPMA10* expression and NaHCO_3_ stress tolerance in grape calli. (a) LUC experiments revealing the enhanced transcriptional activation of *VvPMA10* by VvMYC2 in the presence of VvERF1B. The number in brackets indicates the ratio of *Agrobacterium tumefaciens* containing different expression vectors. (b) Phenotypes of the WT and transgenic calli under normal and 2.5 mm NaHCO_3_ conditions for 21 days. (c–h) Growth (c), MDA content (d), *VvPMA10* expression (e), and PMA activity (f) from the same calli shown in panel b. (g) The oxalate content in the extrudate of calli from the same materials as shown in panel b. (h) H^+^ secretion level. The calli in panel k were treated with 100 mm NaHCO_3_ for 6 h and then were subcultured on medium containing 0.006% bromocresol purple. Different lowercase letters represent significant differences at *P* < 0.05.

*VvERF1B* overexpression did not significantly up‐regulate the expression of *VvMYC2* in roots (Figure S8), excluding the transactivation of *VvMYC2* by VvERF1B. By contrast, yeast two‐hybrid assays (Y2H), BiFC assays, pull‐down assays, and luciferase complementarity assays showed that VvERF1B interacts with VvMYC2 (Figure 6a–d). *Agrobacterium*‐mediated transient expression of the LUC gene in tobacco leaves was performed. The luminescence intensity of leaves co‐transformed with pRI‐101‐AN‐VvERF1B, pGreenII 62‐SK‐VvMYC2, and *VvPMA10‐G‐Box*‐pGreenII 0800‐LUC was markedly increased compared with leaves transformed with pGreenII 62‐SK‐VvMYC2 and *VvPMA10‐G‐Box*‐pGreenII 0800‐LUC (Figure 7a). Therefore, VvERF1B interacted with VvMYC2 and thus increased the transactivation of *VvMYC2*.

To clarify the function of the VvERF1B‐VvMYC2 complex, calli overexpressing both *VvERF1B* and *VvMYC2* and calli overexpressing *VvERF1B* and with mutant *VvMYC2* were obtained (Figure S9). Compared with WT calli, *VvERF1B* or *VvMYC2* overexpression increased NaHCO_3_ stress tolerance based on phenotypic analysis, elevated growth increment and decreased MDA content (Figure 7b–d). NaHCO_3_ stress tolerance was enhanced under simultaneous *VvERF1B* and *VvMYC2* overexpression compared with the overexpression of only *VvERF1B* or *VvMYC2*, and *VvERF1B*‐induced NaHCO_3_ stress tolerance was reduced when *VvERF1B* was overexpressed and *VvMYC2* was mutated (Figure 7b–d). Additionally, simultaneous overexpression of *VvERF1B* and *MYC2* increased *VvPMA10* expression, PMA activity, and oxalate and H^+^ secretion, and the effects of *VvERF1B* overexpression and *VvMYC2* mutation were opposite to those of *VvERF1B* overexpression (Figure 7e–h). Notably, *VvERF1B* overexpression and *VvMYC2* mutation increased NaHCO_3_ stress tolerance but decreased the expression of *VvPMA10* compared with WT calli (Figure 7b–e), suggesting that *VvERF1B* might regulate NaHCO_3_ stress tolerance via the other pathways. Thus, VvERF1B enhanced NaHCO_3_ stress tolerance partially by promoting the VvMYC2‐mediated pathway.

## Discussion

### The VvERF1B‐VvMYC2 module might integrate ethylene and JA signals to regulate NaHCO_3_ stress tolerance

Ethylene and JA play key roles in abiotic stress responses. We clarified the role of ethylene in increasing NaHCO_3_ stress tolerance using exogenous ACC treatment and overexpressing *VvACS3* in grapevines. The content of JA and expression of JA biosynthesis‐related genes were increased by NaHCO_3_ stress (Figure S10a–d). The function of JA in ameliorating the effects of Na_2_CO_3_ or NaHCO_3_ stress was elucidated in grapevines and maize seedlings (Figure S10e–g; Mir *et al*., 2018). Crosstalk between ethylene and JA has been observed under various types of stress. For example, ethylene and JA cooperatively regulate selenite tolerance, and they antagonistically regulate ozone‐induced spreading cell death in *Arabidopsis* (Tamaoki *et al*., 2008; Tuominen *et al*., 2004). Ethylene‐induced *ERF15* and *ERF16* increase the JA level via up‐regulating the expression of the key genes in JA biosynthesis, and JA‐activated MYC2 functions as the transcriptional activators of *ERF16* in tomato leaves (Hu *et al*., 2021). In this study, the expression of *VvACS3* and the JA synthesis‐related genes (*VvAOC*, *VvAOS*, and *VvLOX*) was induced by MeJA and ethylene, respectively (Figure S11a–d), suggesting the interplay between ethylene and JA. Additionally, JA played similar roles as ethylene in regulating the levels of H^+^ and oxalate secretion (Figure S10i,h) and the expression of *VvERF1B*, *VvMYC2*, and *VvPMA10* (Figure S11e–f). Therefore, it is suggested that ethylene and JA might regulate NaHCO_3_ stress tolerance via their interplay.

*Arabidopsis AtERF1* (accession number At3g23240, also known as *AtERF1B* or At‐ERF#092; Zhang *et al*., 2014a) has been shown to increase salt, drought, and heat stress tolerance by integrating JA, ethylene, and ABA signals (Cheng *et al*., 2013). In grapevine, the subgroup of VvERF1 contains VvERF1, VvERF1A, and VvERF1B, and VvERF1 is the most similar to AtERF1, followed by VvERF1B (Figure S12). The high similarity between VvERF1B and AtERF1 and up‐regulation of *VvERF1B* by ethylene and JA (Figure 3a,b; Figure S11e) suggests that *VvERF1B* might play a role in both the ethylene and JA signalling pathways. *VvERF1B* overexpression did not alter the expression of *VvMYC2* (Figure S8); by contrast, the expression of *VvMYC2* was strongly induced by NaHCO_3_ and MeJA treatments (Figure 5d, Figure S11). This indicates that *VvMYC2* transcription might be activated mainly by the JA signal under NaHCO_3_ stress. Apple MdMYC2 up‐regulates the expression of *MdACS1* by activating *MdERF3* transcription and thereby promoting ethylene synthesis (Li *et al*., 2017); AtMYC2 represses AtEIN3 function and affects ethylene signalling in *Arabidopsis* (Zhang *et al*., 2014b). It is suggested that JA might induce the expression of *VvMYC2*, which alters ethylene signalling, under NaHCO_3_ stress.

Therefore, VvERF1B affected VvMYC2 via protein–protein interactions (Figure 6), and VvMYC2 might regulate the expression of *VvERF1B* by affecting ethylene signalling; the VvERF1B‐VvMYC2 module might be a key switch that mediates ethylene‐ and JA‐induced NaHCO_3_ stress tolerance in grapevines.

### A possible mechanism by which ERF1B‐MYC2‐PMA10 regulates NaHCO_3_ stress tolerance

*VvERF1B* and *VvMYC2* were found to play a role in increasing NaHCO_3_ stress tolerance via their overexpression in grapevines, calli, and/or *Arabidopsis* plants (Figures 3 and 5). Mutation of *VvMYC2* partially reduced the effects of *VvERF1B* (Figure 7b–d), suggesting that *VvMYC2* played a role in the *VvERF1B*‐mediated pathway.

Several studies have shown that MYC2 plays various roles via a protein–protein interaction network. For example, the WRKY46‐MYC2 complex plays a key role in promoting anti‐herbivore responses in *Arabidopsis* (Hao *et al*., 2024). Tomato SlERF.F5 interacts with SlMYC2 to cooperatively regulate chlorophyll metabolism (Chen *et al*., 2022). Kazan and Manners (2013) identified 10 proteins that interact with MYC2, and these interactions are involved in cytokinin, GA, JA, and ABA signalling. Several different interaction domains have been reported for MYC2, such as the JID domain, which mediates interactions with JAZ and WRKY46 (Hao *et al*., 2024; Zhang *et al*., 2015), and the TAD domain, which mediates interactions with MED25 (Zu *et al*., 2024). Therefore, VvERF1B is a member of the proteins that interact with MYC2.

The mechanism by which the VvERF1B‐VvMYC2‐VvPMA10 module regulates NaHCO_3_ stress tolerance might be related to the following aspects. First, their interaction enhances MYC2 activity according to our results (Figure 7a) and previous studies showing that the interactions of MYC2 with MED25 or MYL1/MYL2 enhance its transactivation activity (An *et al*., 2017; Ogawa *et al*., 2017). Second, the stability of MYC2 is regulated by post‐translational modifications of ubiquitylation and phosphorylation. The E3 ubiquitin ligase PUB10 and the receptor kinase FERONIA decrease MYC stability (Guo *et al*., 2018; Jung *et al*., 2015). VvERF1B increases the stability of VvMYC2 by competing with interaction sites, which prevents the modification of VvMYC2 by other regulatory proteins. Third, MYC2 orchestrates a downstream transcriptional cascade involving large amounts of genes (Dombrecht *et al*., 2007). It was reported that E‐box (5′‐CAC NTG‐3′) (de Pater *et al*., 1997), G‐box (5′‐CAC GTG‐3′), and G‐box‐related hexamers (Abe *et al*., 1997; Godoy *et al*., 2011; Yadav *et al*., 2005) were the *cis*‐acting sequences bound by MYC2. Here, VvMYC2 specifically bound to the G‐box in the *VvPMA10* promoter, and this binding activity was not observed in other types of binding elements (Figures 5a–c and S3). It is inferred that VvERF1B might promote the binding of VvMYC2 to the G‐box site via protein–protein interactions and *VvPMA10* expression.

The expression of *VvERF1B, VvMYC2*, and *VvPMA10* was strongly induced by NaHCO_3_ (Figure 5d; Xiang *et al*., 2019), and this was observed only under NaHCO_3_ treatment (Figures 3, 4, 5), suggesting that NaHCO_3_ is required to initiate the VvERF1B‐VvMYC2‐VvPMA10 regulatory cascade.

### VvPMA10 might be a key member of the PMA family, which plays a role in increasing NaHCO_3_ stress tolerance in grapevines

PMAs belong to the P‐type subfamily of H^+^‐ATPases. Eleven PMA members have been identified in *Arabidopsis* (Palmgren, 2001). In our study, eight grape PMA genes were predicted by conducting searches of homologous proteins of *Arabidopsis* PMA in the grape genome (Figure 4a). PMAs play key roles in plant growth, development, and responses to various types of stress, including salt and high pH, drought, and heavy metal toxicity (Li *et al*., 2022).

NaHCO_3_ stress induces Na^+^ and ^−^HCO_3_ toxicity, high pH stress, and osmotic stress. To cope with high pH stress, plants need to transport cellular H^+^ to the rhizosphere to maintain a H^+^ gradient across the plasma membrane (membrane potential) (Morth *et al*., 2011). PMAs are the major pump responsible for root H^+^ efflux (Fuglsang *et al*., 2007). *VvPMA10* overexpression increased the H^+^ efflux rate of the roots (Figure 4g) and the H^+^ secretion of calli (Figure 4o); *Arabidopsis* AHA2 and AHA7 and *Sesuvium portulacastrum* SpAHA1 mediate H^+^ efflux in the roots (Fan *et al*., 2018; Yuan *et al*., 2017). Under Na^+^ stress, AHA2‐mediated root H^+^ efflux is essential for the activation of the SOS1 Na^+^/H^+^ antiporter (Yang *et al*., 2019b), and root Na^+^ efflux mediated by the SOS1 Na^+^/H^+^ antiporter is effective for increasing Na^+^ tolerance (Yang and Guo, 2018). VvPMA10 shared high similarity more than 76% to AHA10 and AHA2 (Figure S13), and it might play a similar role in alleviating Na^+^ stress by promoting Na^+^ efflux. Under ^−^HCO_3_ stress, *Arabidopsis* seedling and calli growth are not affected by NaCl stress when concentrations of NaCl are less than 10 mm (Yang *et al*., 2019a, data about calli were not shown); however, leaf chlorosis and lethal symptoms are observed when NaCO_3_ concentrations are less than 10 mm (Ye *et al*., 2019). *VvPMA10* overexpression significantly alleviated 2 and 4 mm NaHCO_3_‐induced growth inhibition in *Arabidopsis* seedlings (Figure S5) and calli (Figure 4j,k), respectively. Thus, *VvPMA10* plays a role in increasing ^−^HCO_3_ stress tolerance. The mechanism by which PMA increases ^−^HCO_3_ stress tolerance remains unclear. Nevertheless, the membrane potential generated by PMA promotes the movement of various anions across the plasma membrane, including NO_3_^−^, H_2_PO_4_^−^/HPO_4_^2−^, SO_4_^2−^, and citrate anions, via transporters (Espen *et al*., 2004; Preuss *et al*., 2010; Takahashi *et al*., 2012; Zhang *et al*., 2017). Hence, *VvPMA10* might promote the efflux of ^−^HCO_3_ and thereby alleviate ^−^HCO_3_ injury.

Anions are needed to maintain an equilibrium following the secretion of H^+^ to the apoplast. Increased oxalate secretion was observed in *VvPMA10*‐overexpressing roots and calli under NaHCO_3_ stress (Figure 4h,o). VvPMA10 might promote oxalate secretion by activating a transporter that has not yet been identified in plants. Moreover, organic acids are often released in response to environmental stress, and these responses are highly stress‐ and plant‐species specific (Jones, 1998). Therefore, VvPMA10 might increase NaHCO_3_ stress tolerance by promoting oxalic acid synthesis. Although the secretion of organic acids alleviates alkaline salt injury by maintaining the pH and ion balance of the roots and rhizosphere (Jia *et al*., 2019; Yang, 2012), additional studies are needed to clarify the role of oxalate in increasing NaHCO_3_ stress tolerance.

Collectively, VvPMA10 plays a crucial role in increasing NaHCO_3_ stress tolerance, and this might be attributed to the increased efflux of H^+^ and oxalate anions.

**Figure 8:**
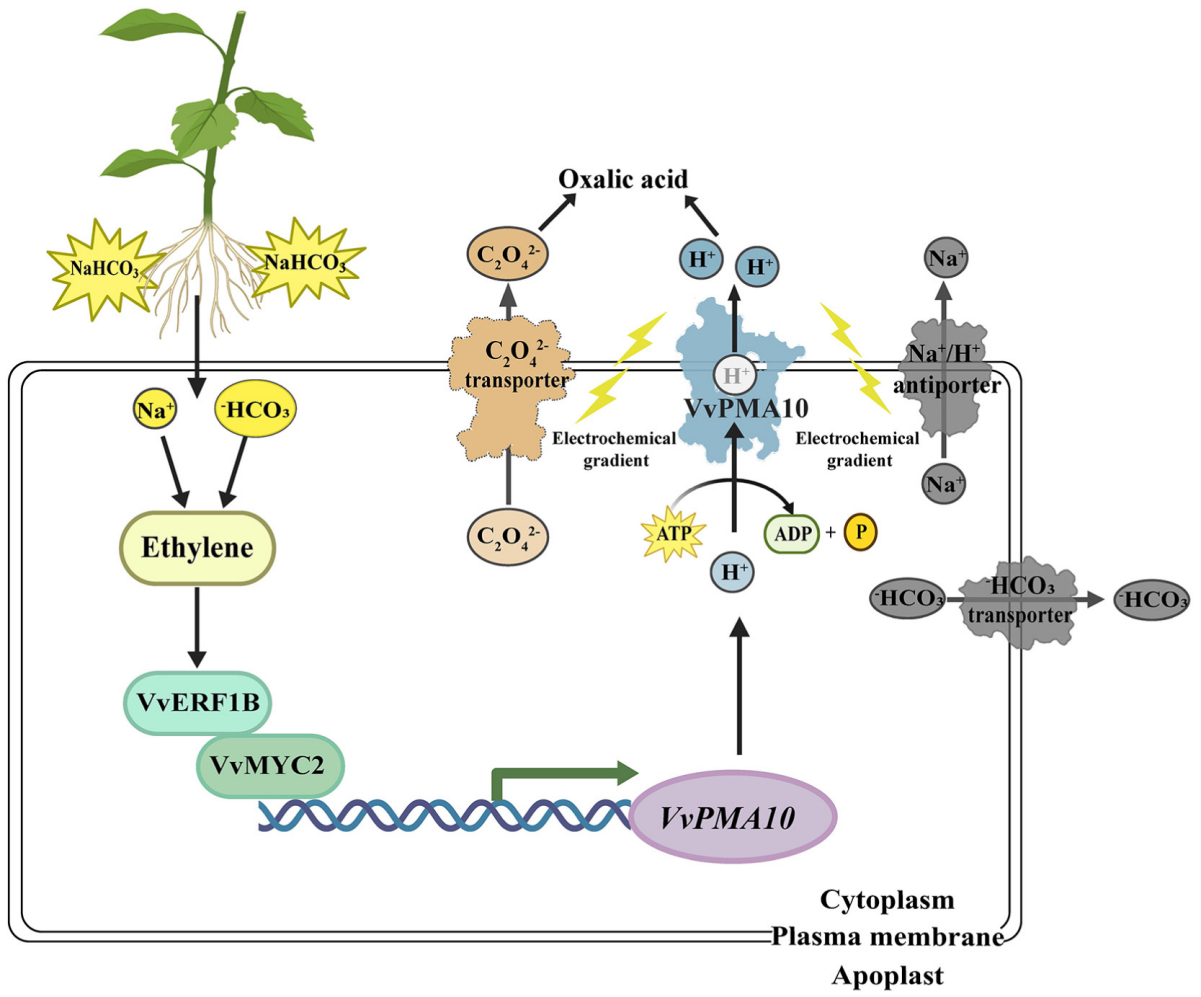
Model of the regulation of NaHCO_3_ stress tolerance by ethylene via the VvERF1B‐VvMYC2‐VvPMA10 pathway. NaHCO_3_ stress induces ethylene production and up‐regulates *VvERF1B* expression. VvERF1B interacts with VvMYC2 to activate *VvPMA10* expression, which increases PMA activity. The enhanced PMA activity promotes H^+^ efflux and generates an electrochemical gradient, which provides energy for the transport of oxalate, Na^+^, and ^−^HCO_3_ across the plasma membrane.

In summary, a working model for the role of the VvERF1B‐VvMYC2‐VvPMA pathway during ethylene‐induced NaHCO_3_ stress tolerance was developed in grapevines (Figure 8). Specifically, ethylene enhances the NaHCO_3_ stress tolerance of grapevines, which is accompanied by increased PMA activity and H^+^ and oxalate secretion. VvERF1B functions downstream of the ethylene signalling pathway and plays a key role in increasing NaHCO_3_ stress tolerance. VvERF1B interacts with VvMYC2 and increases its ability to transactivate *VvPMA10*, which enhances NaHCO_3_ stress tolerance. Additionally, VvPMA10 can promote the secretion of H^+^ and oxalate anions, which can mediate the formation of oxalic acid. The efflux of H^+^ across the plasma membrane generates an electrochemical gradient, which might provide energy for the transport of Na^+^ and ^−^HCO_3_ and thereby reduce NaHCO_3_ injury. NaHCO_3_ stress conditions up‐regulate the VvERF1B‐VvMYC2‐VvPMA pathway.

## Materials and methods

### Plant materials and growth conditions

Grapevine rootstock SA15 cuttings with high NaHCO_3_ stress tolerance were used to evaluate the effects of ACC and 1‐MCP on the NaHCO_3_ stress tolerance of grapevine. The grapevine cuttings were planted in plastic pots (12 cm diameter, 25 cm high) with a substrate‐to‐vermiculite ratio of 2:1. The vines were grown in a greenhouse under natural conditions. The *in vitro* shoot cultures of SA15 were used to measure H^+^ secretion via bromocresol purple and *Agrobacterium tumefaciens*‐mediated root transformation. The shoot cultures were subcultured in MS medium with 0.2 mg/L indolebutyric acid (IBA) under the following conditions: 25 °C, 16 h/8 h light/dark photoperiod, and 600 μmol/m^2^/s light intensity.

‘Red Gamay’ grape calli were used to obtain transgenic calli. The calli were subcultured in B5 medium containing 30 g/L sucrose, 0.5 g/L casein hydrolysate, 0.5 mg/L kinetin, and 2 g/L phytagel (pH 5.8–6.0) at 25 °C. Embryogenic ‘Thompson Seedless’ calli were used to obtain transgenic grapevines via genetic transformation. They were subcultured on MS medium containing 3 g/L phytagel, 45 g/L sucrose, and 1.2 g/L activated charcoal.

### Determination of the MDA content, relative conductivity, root activity, oxalate content, ethylene production rate, and JA content

The MDA content, relative conductivity, and root activity were determined using previously described methods (Xu *et al*., 2019).

For oxalate extraction, the MS medium was lyophilized and extracted with 80% methanol three times. The extract was then concentrated, and the supernatant was collected and filtrated through a 0.45‐μm filter. The filtrate was used to determine the content of oxalate using a capillary electrophoresis system (Beckman P/ACE, Palo Alto, CA) (Xiang *et al*., 2019). The oxalate content was expressed according to the ratio of its amount in MS medium to the weight of roots or calli.

The ethylene production rate was determined using a Shimadzu GC‐9A gas chromatograph (Kyoto, Japan; Xiang *et al*., 2019). Extraction and determination of JA were performed according to our previous study (Lv *et al*., 2021).

### Measurement of PMA activity, H^+^ flux, and H^+^ secretion

The plasma membrane proteins from grapevine roots and calli were isolated using sucrose density gradient centrifugation (Yan *et al*., 2002). Five milligrams of these plasma membrane proteins were then added to a 5 mL reaction solution containing 5 mm MgSO₄, 1 mm Na₂MoO₄, 5 mm NaN₃, 5 mm ATP, 50 mm KCl, 0.2% Brij 58, and 30 mm BTP/MES buffer (pH 6.5). The mixture was incubated for 30 min before the reaction was terminated with 1 mL of stop solution (2% H₂SO₄, 5% SDS, and 0.7% (NH₄)₂MoO₄). H^+^‐ATPase activity was then calculated by measuring the amount of inorganic phosphate (Pi) released, at 820 nm using a spectrophotometer (Yan *et al*., 2002).

The H^+^ fluxes of grapevine roots were measured using the scanning ion‐selective electrode technique with a Noninvasive Microtest Technology (NMT) system (Xuyue Beijing Sci. & Tech. Co., Ltd., Beijing, China; Xiang *et al*., 2019). Before measuring the ion fluxes, grapevine roots were secured in a culture dish and equilibrated in a testing buffer (0.1 mm KCl, 0.1 mm CaCl_2_, 0.1 mm MgCl_2_, 0.5 mm NaCl, 0.2 mm Na_2_SO_4_ and 0.3 mm MES) for 10 min. Near the root surface, the microelectrode was moved horizontally between two points spaced 30 μm apart. The software recorded the potential difference generated between these points and calculated the H^+^ flux.

H^+^ secretion of grape roots and calli was measured using a previously described method (Xiang *et al*., 2019). One part of the roots of grapevine shoot cultures was covered by a solid medium (pH 5.8) containing 0.006% bromocresol purple (pH indicator, discoloration range of 5.2–6.8), and another part was covered by a solid medium containing 100 mm NaHCO_3_ (pH 8.7); the roots were pretreated with 50 μm ACC for 3 h before they were covered in Petri dishes. Calli were placed on MS medium containing 100 mm NaHCO_3_ for 6 h and then transferred to medium with bromocresol purple.

### Genetic transformation of grapevines, vine roots, calli, and *Arabidopsis*

The full‐length coding sequences (CDSs) of *VvERF1B*, *VvMYC2*, and *VvPMA10* were cloned into the pRI‐101‐AN‐GFP vectors with kanamycin tolerance and pHB‐GFP vector (only for *VvMYC2*) with hygromycin tolerance for overexpression. Four protospacer adjacent motif (PAM) sequences in the exons of *VvERF1B* and *VvMYC2* were designed using the online tool CRISPRdirect (https://crispr.dbcls.jp). The PAM sequences were ligated to the helper plasmid (PYLsgRNA‐AtU6‐1), and then the constructs were inserted into the pYLCRISPR/Cas9P35S‐GFP vector to generate the CRISPR/Cas construct (Wang *et al*., 2018).

The pRI‐101‐AN‐GFP vectors containing *VvERF1B* were transformed into embryogenic Thompson Seedless calli using the *Agrobacterium* (LB4404)‐mediated method (Zhang *et al*., 2023b). The pRI‐101‐AN‐GFP vectors containing *VvERF1B*, *VvMYC2*, and *VvPMA10* were transformed into ‘Red Gammy’ calli using the *Agrobacterium* (LB4404)‐mediated method (Zhang *et al*., 2023b) and/or *Arabidopsis* plants using the *Agrobacterium* (GV3101)‐mediated floral‐dip method (Zhang *et al*., 2006). The pHB‐GFP vector containing VvMYC2 was transformed into *VvERF1B*‐overexpressing calli to generate calli overexpressing *VvERF1B* and *VvMYC2*. The pRI‐101‐AN‐GFP vectors containing *VvERF1B* were transformed into grapevine roots using *Agrobacterium rhizogenes* (MSU440)‐mediated transformation (Gao *et al*., 2023). The CRISPR/Cas construct containing *VvERF1B* and *VvMYC2* was transformed into grape calli using the *Agrobacterium* (LB4404)‐mediated method. The CRISPR/Cas construct containing *VvMYC2* was transformed into *VvERF1B*‐overexpressing calli to generate calli overexpressing *VvERF1B* and *VvMYC2* mutants.

### Yeast one‐hybrid assay

The cDNA library of root mRNA was constructed using a CloneMiner II cDNA Library Construction Kit (Invitrogen, Carlsbad) and assembled by Oebiotech Biomedical Technology Co., Ltd. (Shanghai). A 2‐kb sequence upstream of *VvPMA10* was cloned into the pHIS2 vector and used as a bait plasmid. The bait plasmid was co‐expressed with the cDNA library in Y187 yeast to identify transcriptional regulators of *VvPMA10*. Sequences containing the *cis*‐elements (*ERF*, *MYC*, *G‐BOX*, and *E‐BOX*) were cloned into the pHIS2 vector, and the CDSs of *VvERF1B* and *VvMYC2* were cloned into the pGADT7 vector. The pairs of recombinant vectors were co‐transformed into the yeast strain Y187. The interactions were examined using SD/−Trp‐His‐Leu medium containing an appropriate concentration of 3‐amino‐1,2,4‐triazole (3‐AT). The primers used are listed in Table S1.

### Electrophoretic mobility shift assay

*VvERF1B* and *VvMYC2* CDSs were cloned into the His‐tag‐containing pET‐32a vector and incorporated into BL21 (DE3) cells to express fusion proteins. The fusion proteins were purified using His‐tagged BeaverBeads™ IDA‐Nickel (Beaver, BioBay, China). The DNA probes containing the above elements were synthesized with biotin labelling. An EMSA kit (Thermo Fisher Scientific, MA) was used to perform EMSAs.

### Dual‐luciferase assay

*VvERF1B* and *VvMYC2* CDSs were cloned into the pGreenII 62‐SK vector, and the *cis*‐elements in the *VvPMA10* promoter were cloned into the pGreenII 0800‐LUC vector (Zhang *et al*., 2023b). Tobacco leaves (*Nicotiana tabacum*) were transiently transformed with *Agrobacterium tumefaciens* strain GV3101 with the above vectors. The LUC signal was observed using a Chemiluminescent Imaging System (Tanon‐5200) following 2 days of co‐cultivation. The primers used are listed in Table S1.

### Yeast two‐hybrid assay

The CDS of *VvERF1B* was cloned into the *pGADT7* vector, and the *VvMYC2* CDS was cloned into the *pGBKT7* vector. The recombinant plasmids were simultaneously transformed into yeast strain Y2H and incubated on SD medium (−Trp/−Leu/–His/−Ade) for 3–5 days. Positive yeast cells were observed on SD (−Trp/− Leu/–His/−Ade) medium containing X‐α‐Gal.

### Pull‐down assay

The full‐length *VvERF1B* and *VvMYC2* coding sequences were inserted into the pET‐32a and pGEX‐4 T‐1 vectors, respectively. The VvERF1B‐His and VvMYC2‐GST fusion proteins were obtained by expressing them in BL21 (DE3) cells, and VvERF1B‐His was purified via the His‐labelled Protein Purification Kit (Beaver, BioBay, China). Western blotting using anti‐GST and anti‐HIS antibodies (Abmart, Shanghai, China) was performed to identify elution products. GST protein was used as a negative control.

### Bimolecular fluorescence complementation (BiFC) assay

Full‐length *VvERF1B* and *VvMYC2* coding sequences were cloned into the pSPYCE and pSPYNE vectors, respectively, which were transformed into *A. tumefaciens* strain GV3101. The recombinant plasmids in *A. tumefaciens* were transformed into tobacco leaves via injection. Following 3 days of co‐cultivation, fluorescence imaging was conducted under a 20× objective lens using a laser confocal microscope LSM880 (Zeiss, Oberkochen, Germany) following a previously described method.

### Split‐luciferase assay (Split‐LUC)

LUC complementation experiments were conducted to verify protein–protein interactions following a previously described method (Jiang *et al*., 2021). The CDSs of *VvERF1B* and *VvMYC2* were cloned into the pCMABIA1300‐N‐luc and pCMABIA1300‐C‐luc vectors, respectively. The vectors were transferred into *Agrobacterium tumefaciens* strain GV3101, which was then infiltrated into *N. benthamiana* leaves. The LUC signal was observed using a Chemiluminescent Imaging System (Tanon‐5200) following 2 days of infiltration. The primers used in the LUC assays are listed in Table S1.

### Real‐time quantitative PCR (RT‐qPCR)

Total RNA was extracted using the Total RNA Rapid Extraction Kit (Zomanbio, Beijing, China), and the extracted RNA was used to generate first‐strand cDNA using the PrimeScript RT kit (TaKaRa, Dalian, China). RT‐qPCR analysis was performed using SYBR Premix Ex Taq II (Takara, Dalian, China), and the primers are listed in Table S1. Three replications were performed for all experiments.

### Statistical analysis

SPSS (v19.0) software was used to perform analysis of variance and *t*‐tests. Adobe Photoshop and Adobe Illustrator software were used to make figures.

## Author contributions

Guangqing Xiang: Conceptualization, Data curation, Investigation, Writing – original draft preparation. Zongbao Fan: Investigation, Methodology. Shuxia Lan and Dezheng Wei: Resources, Investigation, Visualization. Yazhe Gao and Hui Kang: Investigation, Methodology. Yuxin Yao: Conceptualization, Funding acquisition, Writing – review and editing. All authors have read and agreed to the published version of the manuscript.

## Conflict of interest

The authors declare that they have no known competing financial interests or personal relationships.

## Supporting information


**Figure S1** Identification of *VvERF1B*‐overexpressing and mutant transgenic grape calli.
**Figure S2** Functional identification of *VvERF1B* in *Arabidopsis* plants.
**Figure S3** The experiments for detecting the binding of VvERF1B to the *VvPMA10* promoter.
**Figure S4** CRISPR/Cas9‐mediated mutation of *VvPMA10*.
**Figure S5** Functional identification of *VvPMA10* in *Arabidopsis* plants.
**Figure S6** Y1H experiments for detecting the binding of VvMYC2 to *cis*‐elements in the *VvPMA10* promoter.
**Figure S7** CRISPR/Cas9‐mediated mutation of *VvMYC2*.
**Figure S8** Expression analysis of *VvMYC2* in *VvERF1B* transgenic calli (a) and roots (b).
**Figure S9** Identification of transgenic grape calli with altered *VvERF1B* and *VvMYC2* expression or mutant *VvMYC2*.
**Figure S10** Effects of the exogenous application of JA on the NaHCO_3_ stress tolerance of grapevines.
**Figure S11** Expression analysis of the JA synthesis‐related genes (a‐c), *VvACS3* (d), *VvERF1B* (e), *VvMYC2* (f), and *VvPMA10* (g) in grapevine roots treated with ethylene or MeJA for 6 h.
**Figure S12** The phylogenetic relationships of ERF1 proteins from *Arabidopsis thaliana* and grapevine.
**Figure S13** Alignment of the deduced amino acid sequence of plasma membrane H^+^‐ATPases from *Arabidopsis thaliana* (AHA) and grapevine (PMA).

**Table S1** Primer sequences used in this study.

## Data Availability

All data generated or analysed during this study are included in this published article and its supplementary information files.
