# Supplementary material for: Ethylene increases the NaHCO3 stress tolerance of grapevines partially via the VvERF1B‐VvMYC2‐VvPMA10 pathway
Source: Plant Biotechnol J. 2025 Jan 7;23(4):1076–90. doi: 10.1111/pbi.14565 (PMC11933843; doi:10.1111/pbi.14565)

**Figure S1** Identification of *VvERF1B*-overexpressing and mutant transgenic grape calli.


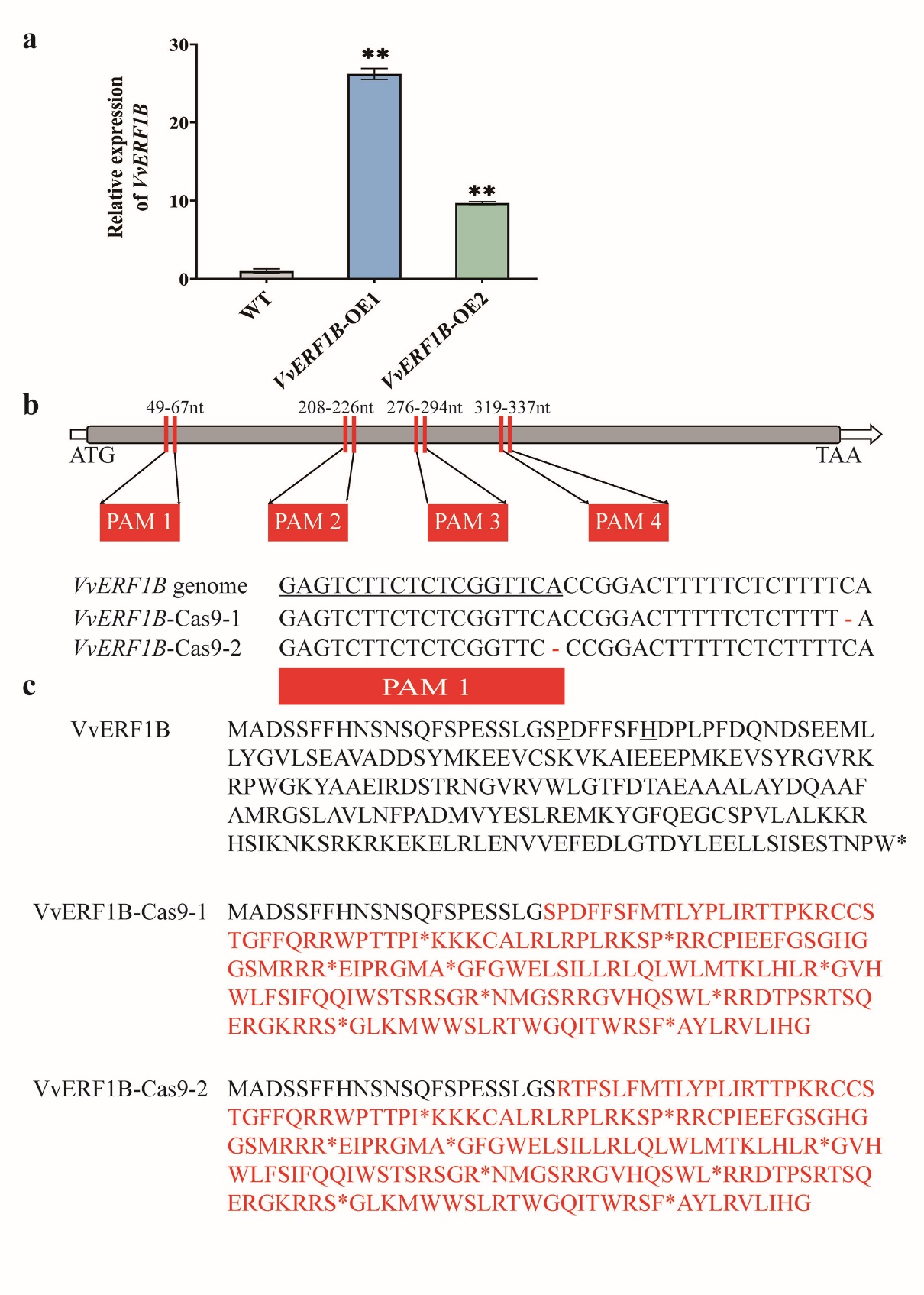


(a) Identification of *VvERF1B*-overexpressing calli using qRT-PCR. (b, c) CRISPR/Cas9-mediated mutation of *VvERF1B*. Panel b and c show changes at the genome and protein levels, respectively. Protospacer adjacent motif, PAM sequence.

**Figure S2** Functional identification of *VvERF1B* in *Arabidopsis* plants.


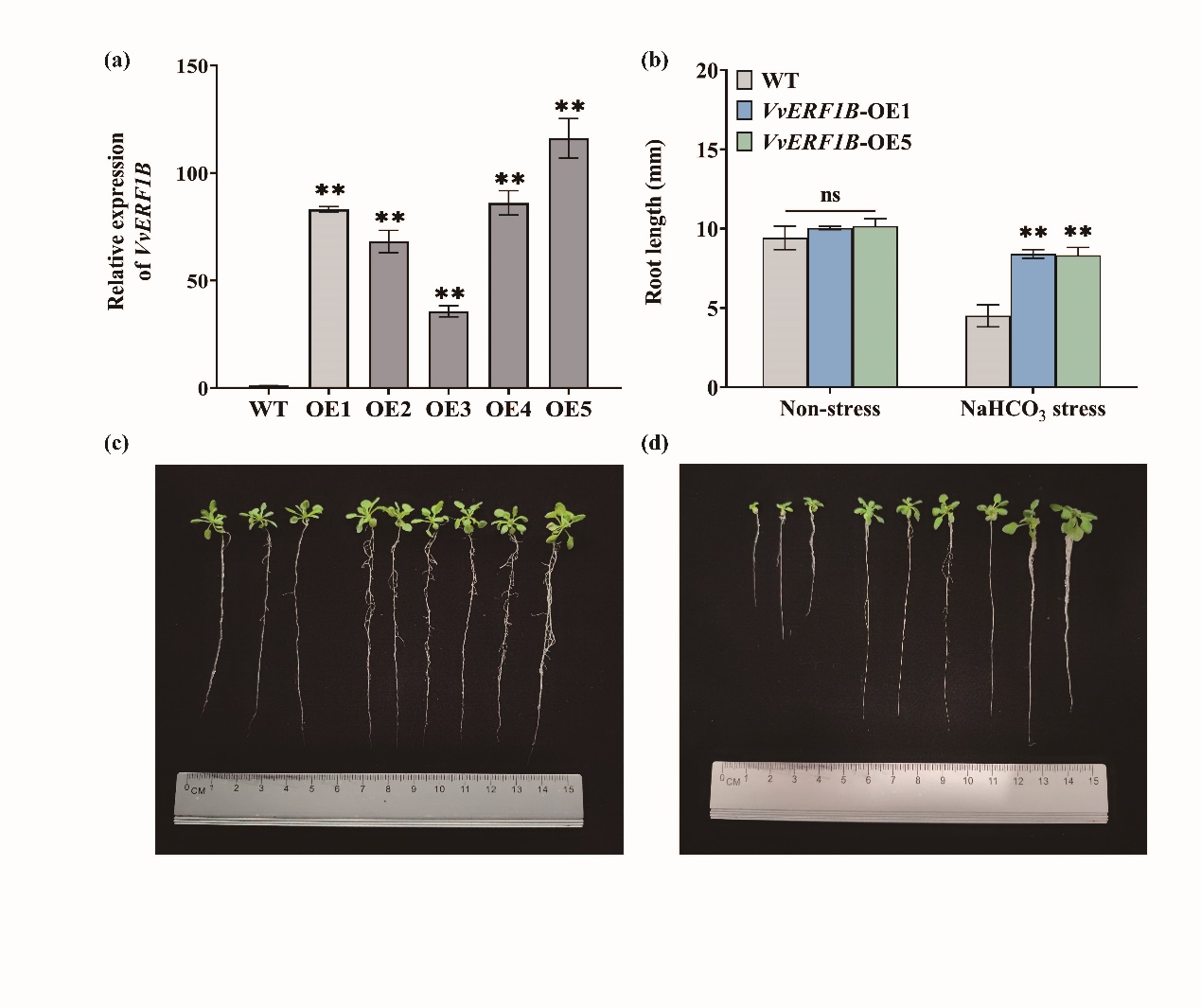


Identification of *VvERF1B*-overexpressing grape calli using qRT-PCR (a). The root length (b) and phenotypes (c, d) of transgenic and WT *Arabidopsis* plants under normal (non-stress) and NaHCO_3_ stress conditions. Significant differences between WT and transgenic lines were calculated using Student'st‐test, **, P‐value <0.01.

**Figure S3** The experiments for detecting the binding of VvERF1B to the *VvPMA10* promoter.


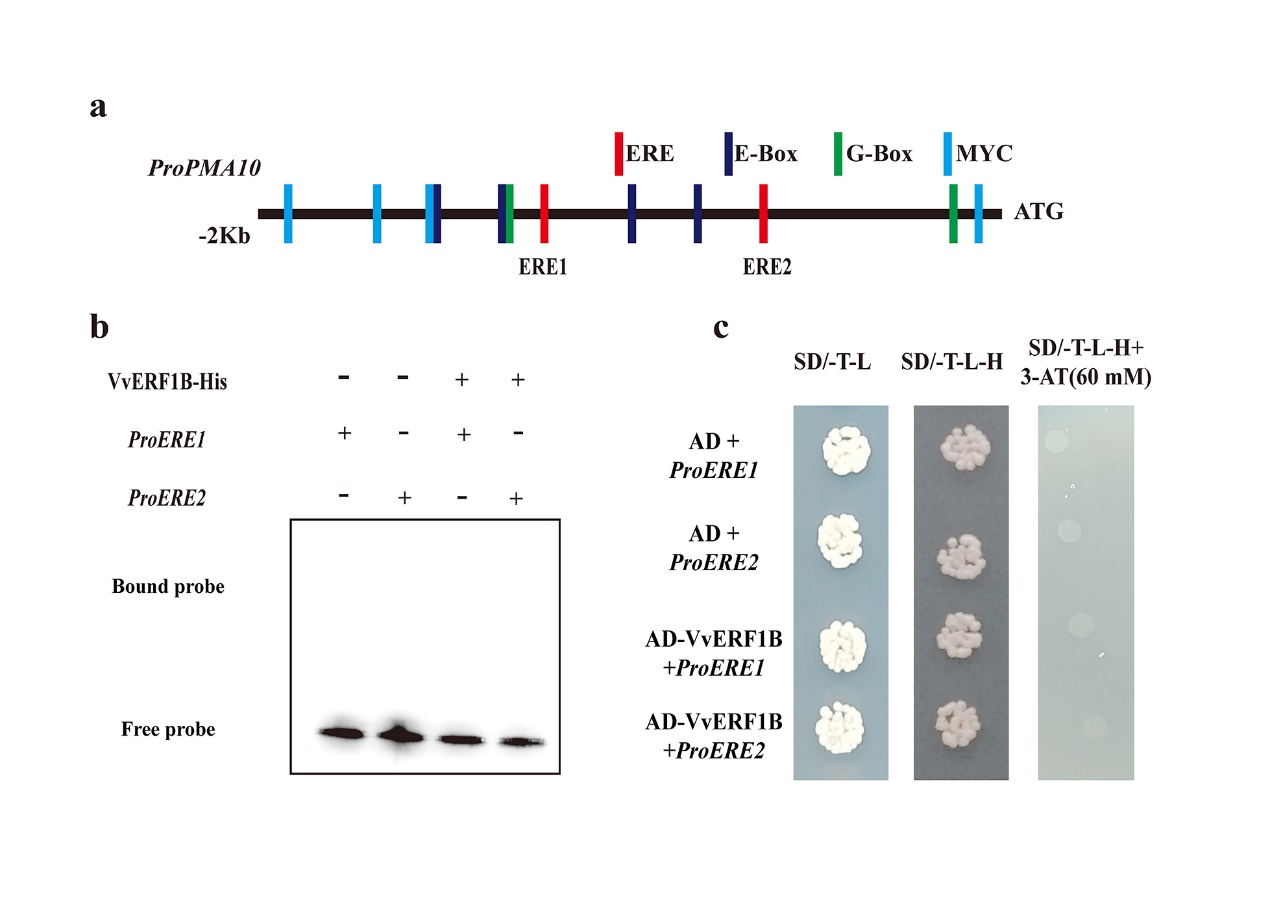


(a) The schematic diagram showing the ERF *cis*-acting elements in the promoter of *VvPMA10*. (b) EMSA experiment. ERE1: TTTAAAAT, ERE2: ATTTTAAA. ‘+’ and ‘-’ indicate presence and absence, respectively, of the indicated probe or protein. (c) Y1H experiment. 3-AT, 3-amino-1,2,4-triazole.

**Figure S4** CRISPR/Cas9-mediated mutation of *VvPMA10*.


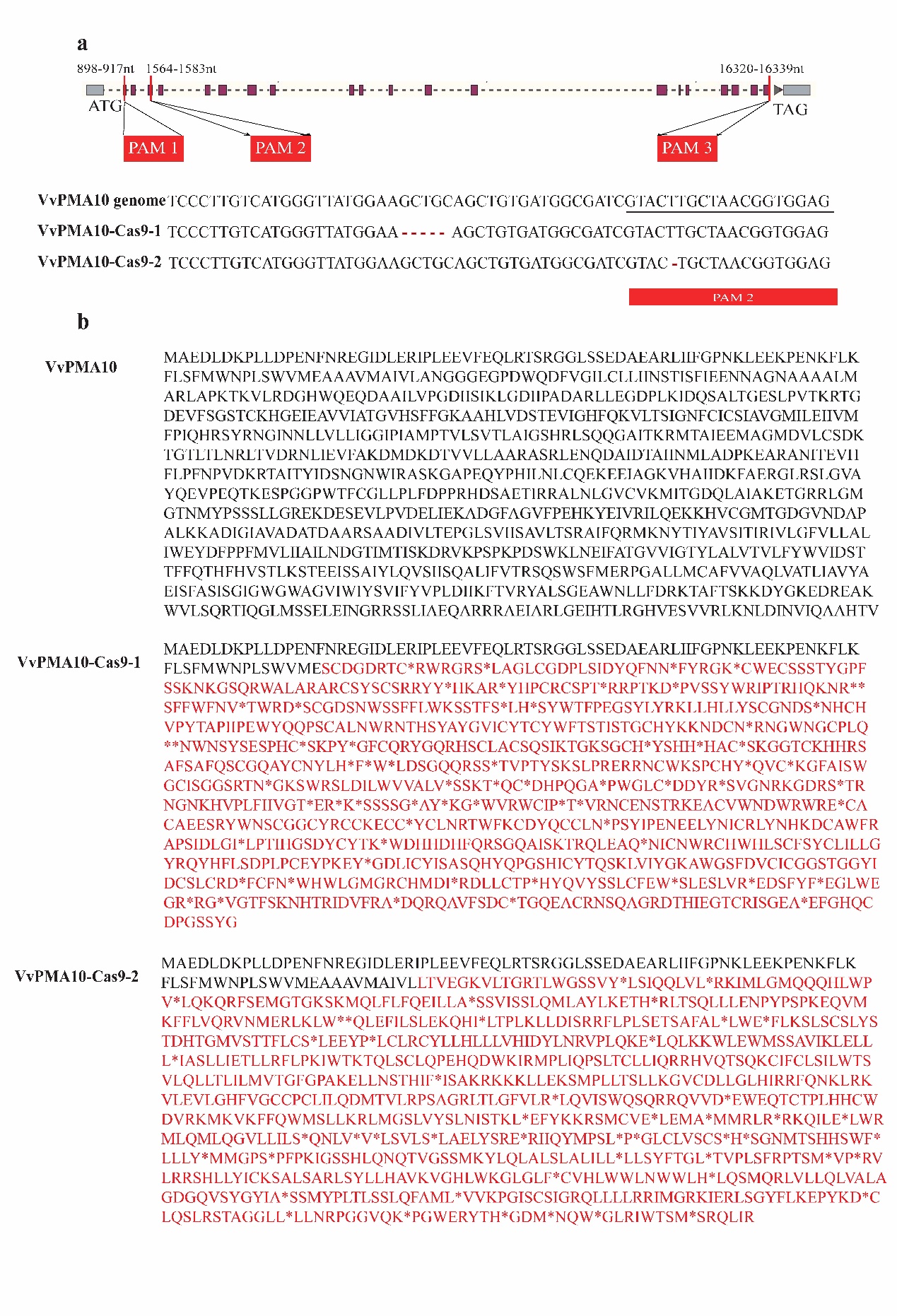


Panel a and b show changes at the genome and protein levels, respectively.

**Figure S5** Functional identification of *VvPMA10* in *Arabidopsis* plants.


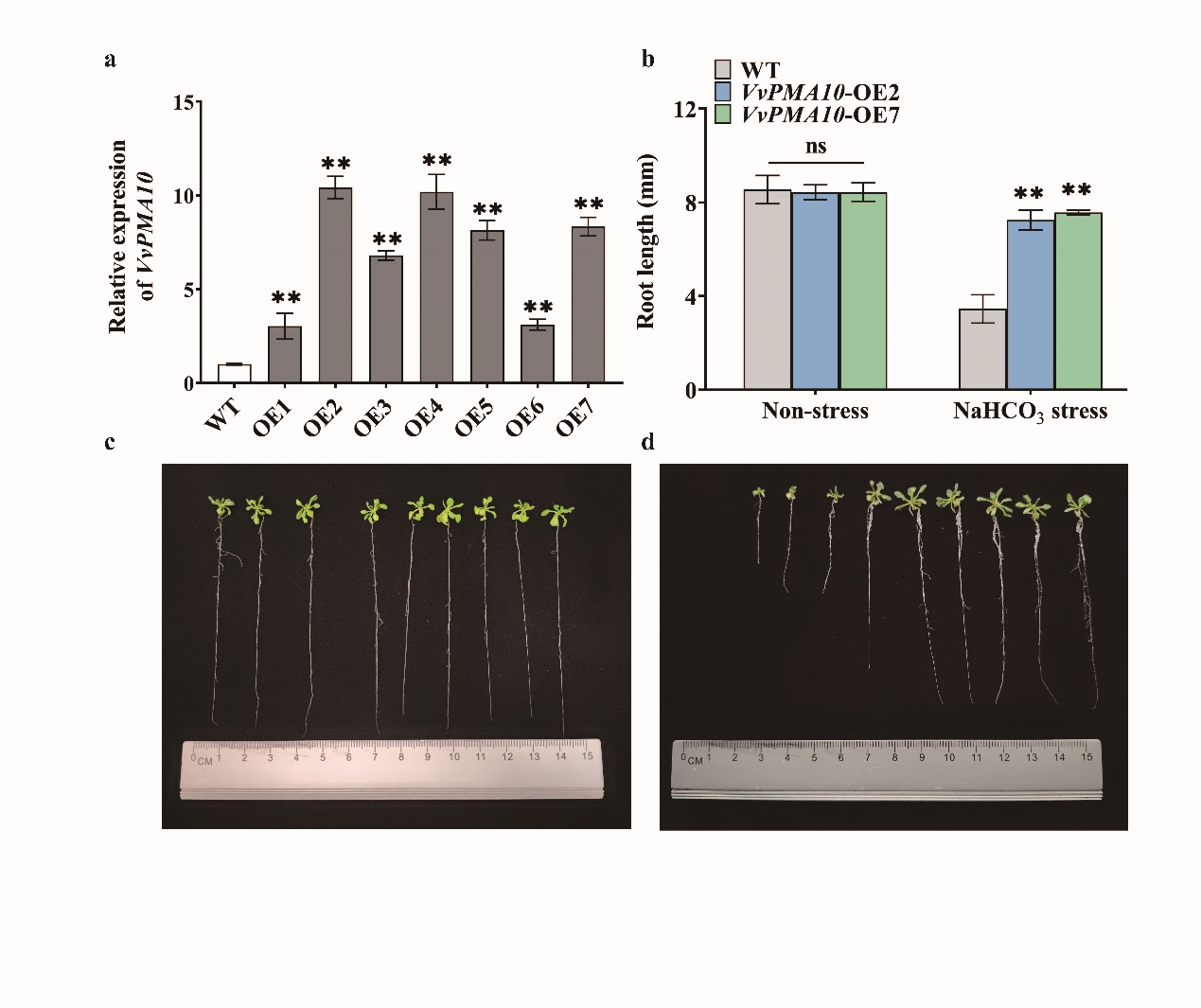


Expression of *VvPMA10* in *A*rabidopsis plants (a). The root length (b) and phenotypes (c, d) of transgenic and WT *Arabidopsis* plants under normal (non-stress) and NaHCO_3_ conditions. Data represent means ± SD (n=3) of three biologically independent experiments. Significant differences between WT and transgenic lines were calculated using Student's t‐test, **, P‐value <0.01.

**Figure S6** Y1H experiments for detecting the binding of VvMYC2 to *cis*-elements in the *VvPMA10* promoter.


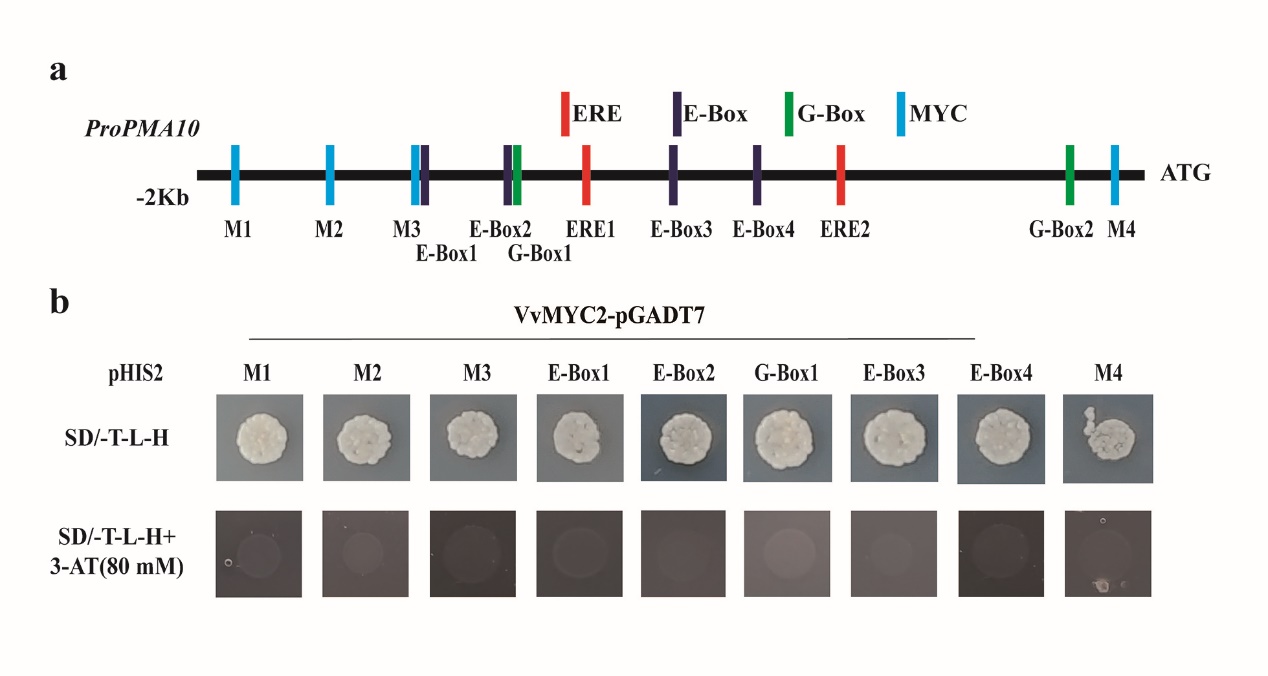


(a) A schematic diagram showing the *cis*-acting elements in the promoter of *VvPMA10*. (b) Y1H. M1, MYC element 1 (CATGTG), M2, MYC element 2 (CATTTG), M3, MYC element 3 (CATGTG), E-Box1, E-Box element 1 (CAGTTG), E-Box2, E-Box element 2 (CACTTG), G-Box1, G-Box element 1 (CACGTC), E-Box3, E-Box element 3 (CATATG), E-Box4, E-Box element 4 (CATATG), M4, MYC element 4 (CAATTG).

**Figure S7** CRISPR/Cas9-mediated mutation of *VvMYC2*.


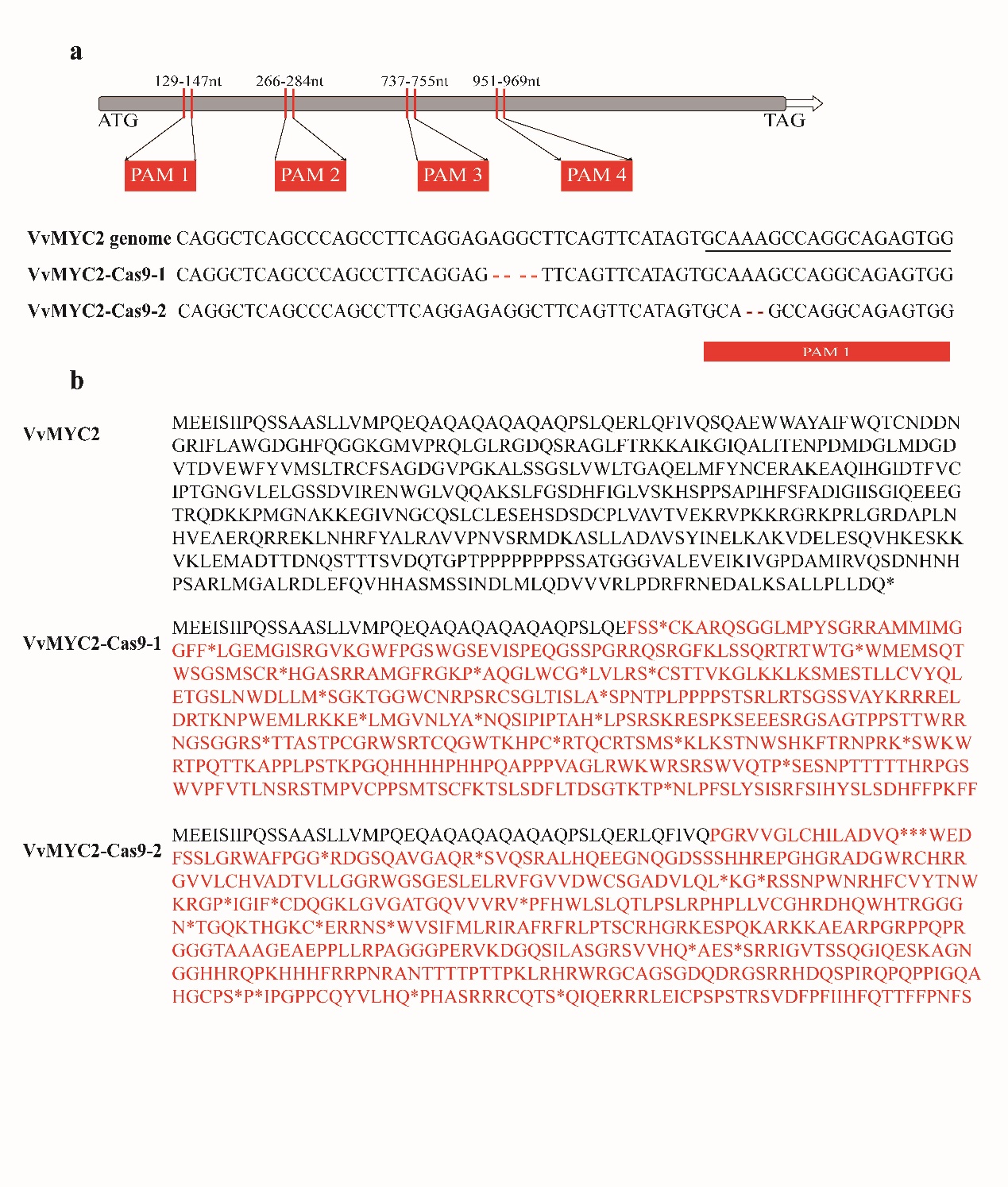


Panel a and b show changes at the genome and protein levels, respectively.

**Figure S8** Expression analysis of *VvMYC2* in *VvERF1B* transgenic calli (a) and roots (b).
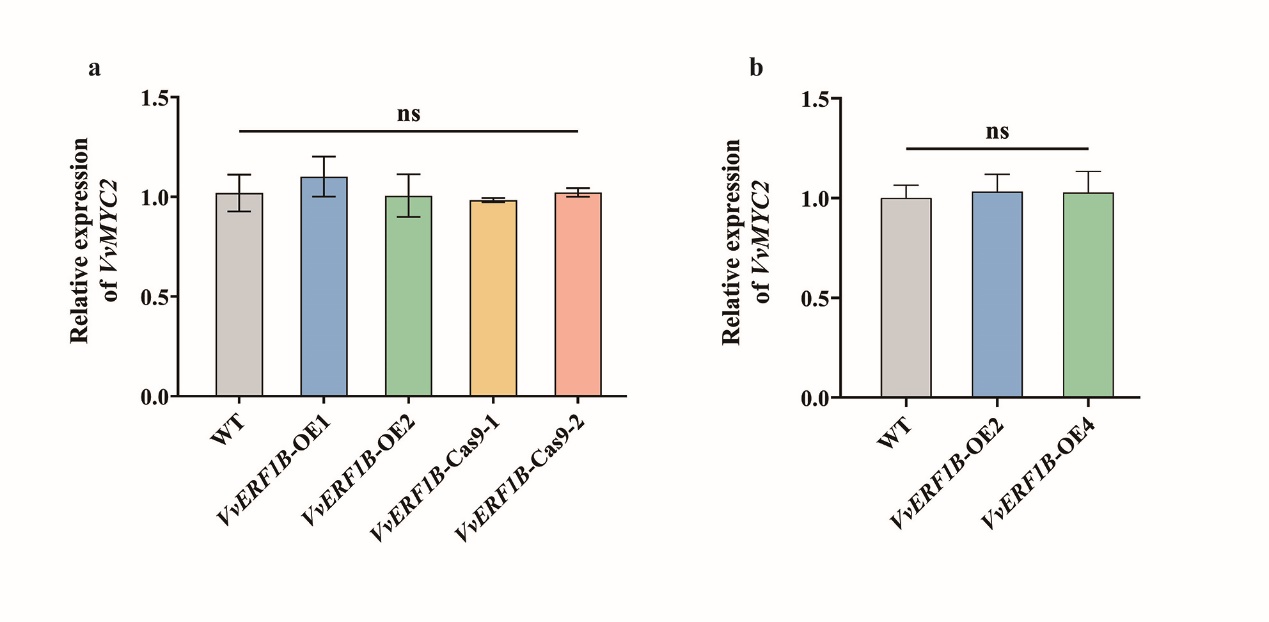


**Figure S9** Identification of transgenic grape calli with altered *VvERF1B* and *VvMYC2* expression or mutant *VvMYC2*.


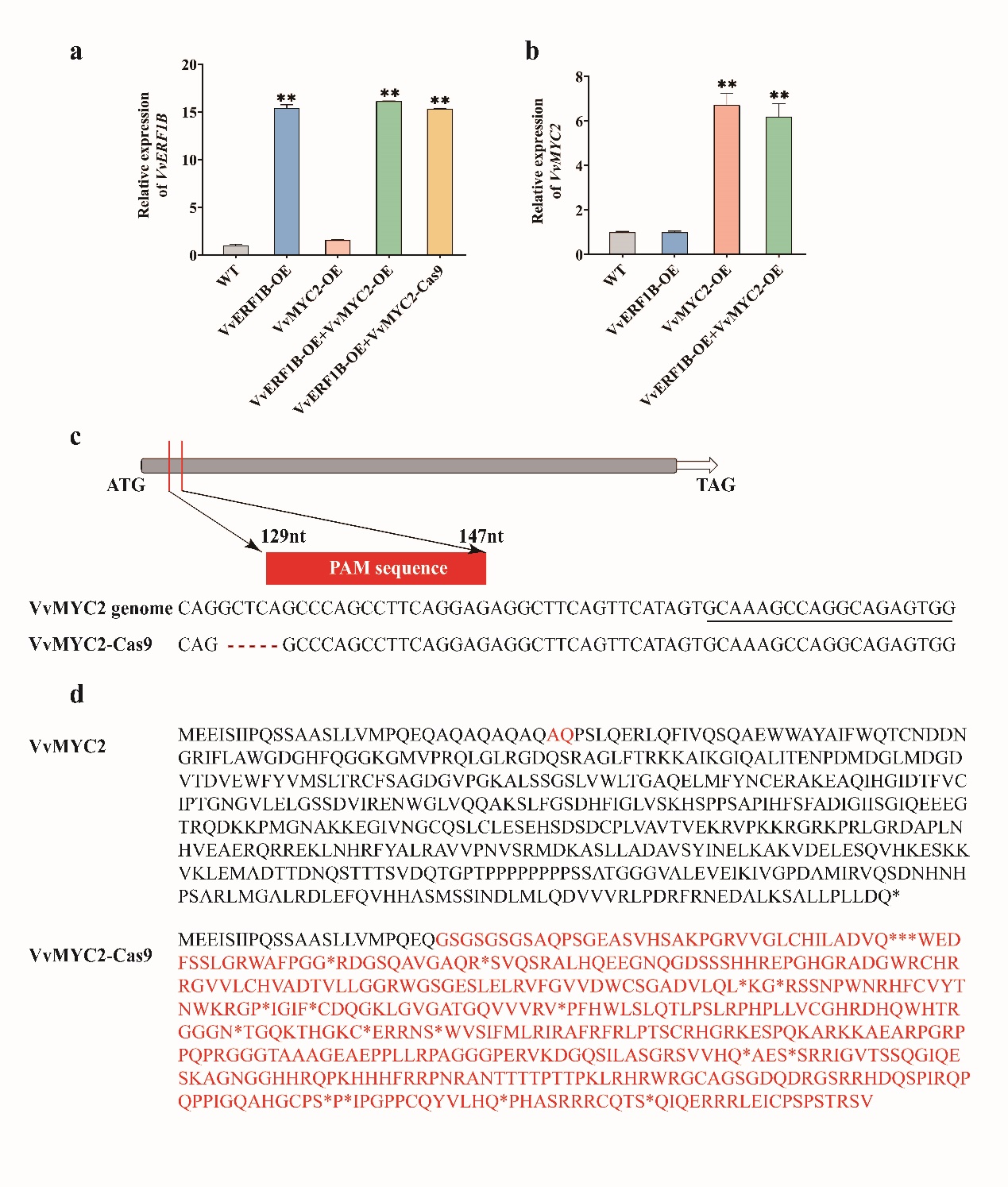


(a) Expression of *VvERF1B* in WT calli, calli overexpressing *VvERF1B*, calli overexpressing *VvMYC2*, calli overexpressing both *VvERF1B* and *VvMYC2*, and calli overexpressing *VvERF1B* and with mutant *VvMYC2*. (b) Expression of *VvMYC2* in WT calli, calli overexpressing *VvERF1B*, calli overexpressing *VvMYC2*, and calli overexpressing *VvERF1B* and with mutant *VvMYC2*. (c, d) CRISPR/Cas9-mediated mutation of VvMYC2. Panel b and c show changes at the genome and protein levels, respectively.

**Figure S10** Effects of the exogenous application of JA on the NaHCO_3_ stress tolerance of grapevines.

**
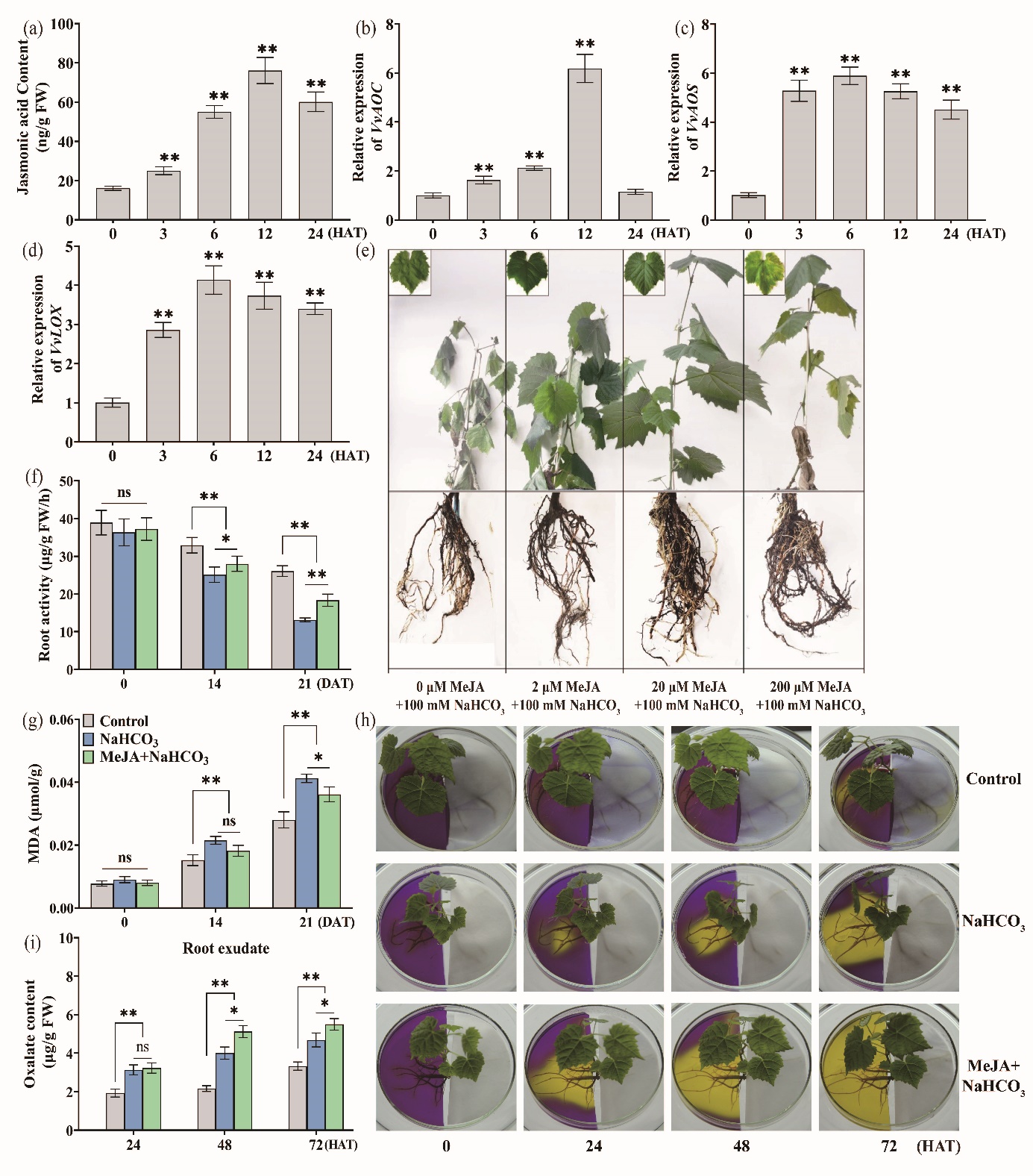
**

JA content (a) and expression of JA synthesis‐related genes (*VvAOC*, *VvAOS* and *VvLOX*) (b-d) in grapevine roots treated with 100 mM NaHCO_3_. (e) Phenotypes of grapevines grown in a greenhouse under various treatments for 21 DAT. Root activity (f), MDA content (g), H^+^ secretion (h) and oxalate content (i) of grapevines under various treatments. Values represent the means ± SD of three replicates. * Significant difference, P < 0.05; ** highly significant difference, P < 0.01.

Compared with the control, NaHCO_3_ stress also significantly increased the content of JA and expression of JA synthesis‐related genes, including *VvAOC*, *VvAOS,* and *VvLOX*, in grapevine roots (Figure S10 a-d). To clarify the role of JA in regulating NaHCO_3_ stress tolerance, grape cuttings were treated with NaHCO_3_ and MeJA for 0, 14, and 21 DAT. NaHCO_3_ stress resulted in leaf withering at 21 d DAT; by contrast, MeJA application alleviated NaHCO_3_ injury (Figure S10 e). MeJA application also significantly increased root activity and reduced the MDA content (Figure S10 f, g). In addition, MeJA application increased the H^+^ secretion level and oxalate content (Figure S10 h, i). Therefore, JA increased the NaHCO_3_ stress tolerance and H^+^ and oxalate secretion of grapevines, which might be attributed to the upregulation in the expression of *VvERF1B*, *VvMYC2*, and *VvPMA10*.

**Figure S11** Expression analysis of the JA synthesis-related genes (a-c), *VvACS3* (d), *VvERF1B* (e), *VvMYC2* (f), and *VvPMA10* (g) in grapevine roots treated with ethylene or MeJA for 6 h.

**
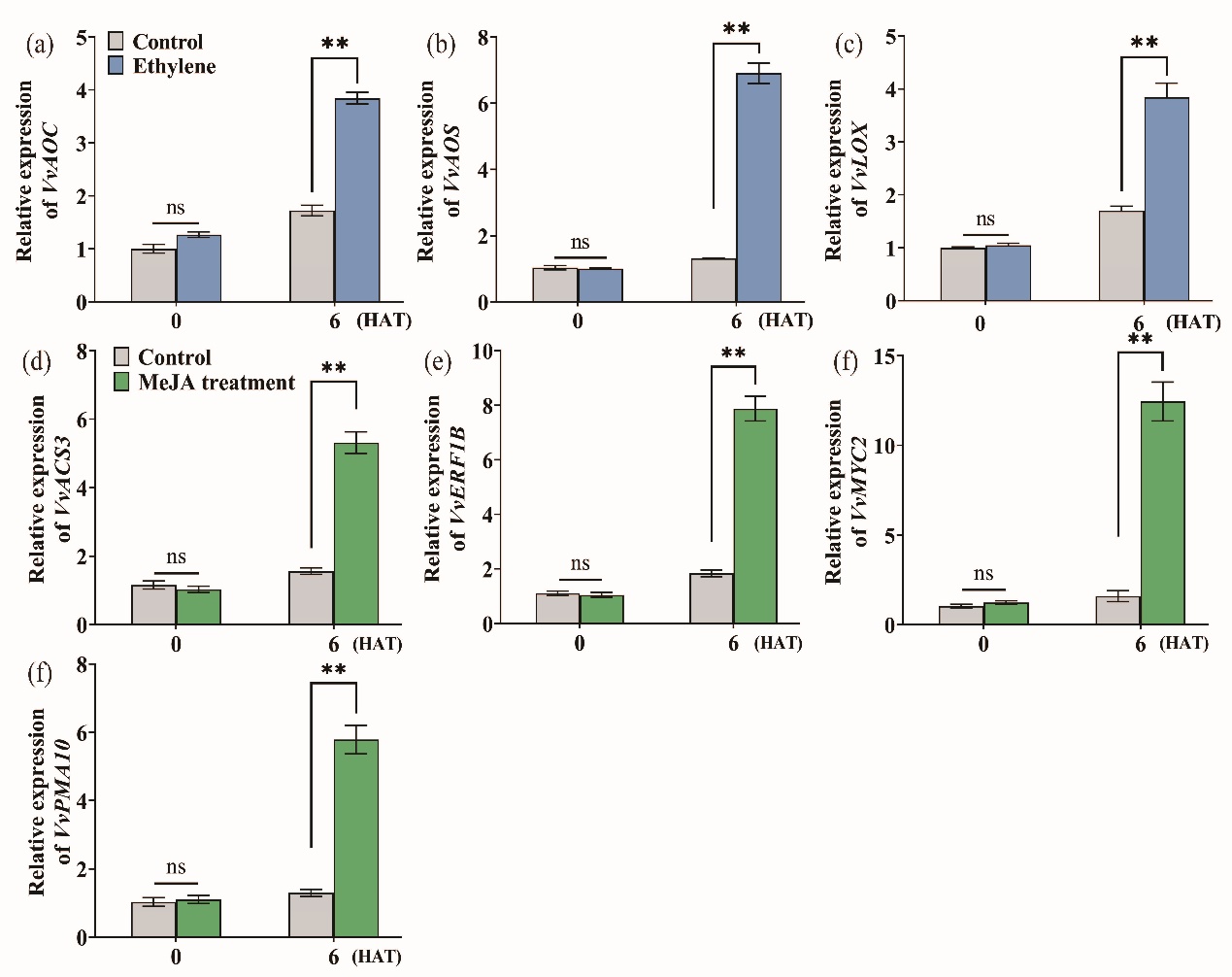
**

**Figure S12** The phylogenetic relationships of ERF1 proteins from *Arabidopsis thaliana* and grapevine.


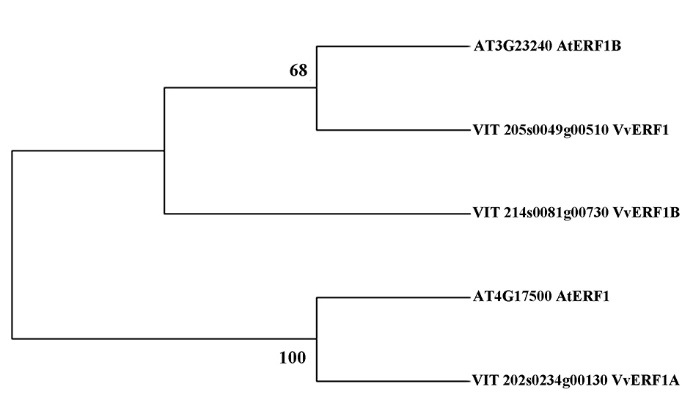


**Figure S13** Alignment of the deduced amino acid sequence of plasma membrane H^+^-ATPases from *Arabidopsis thaliana* (AHA) and grapevine (PMA).


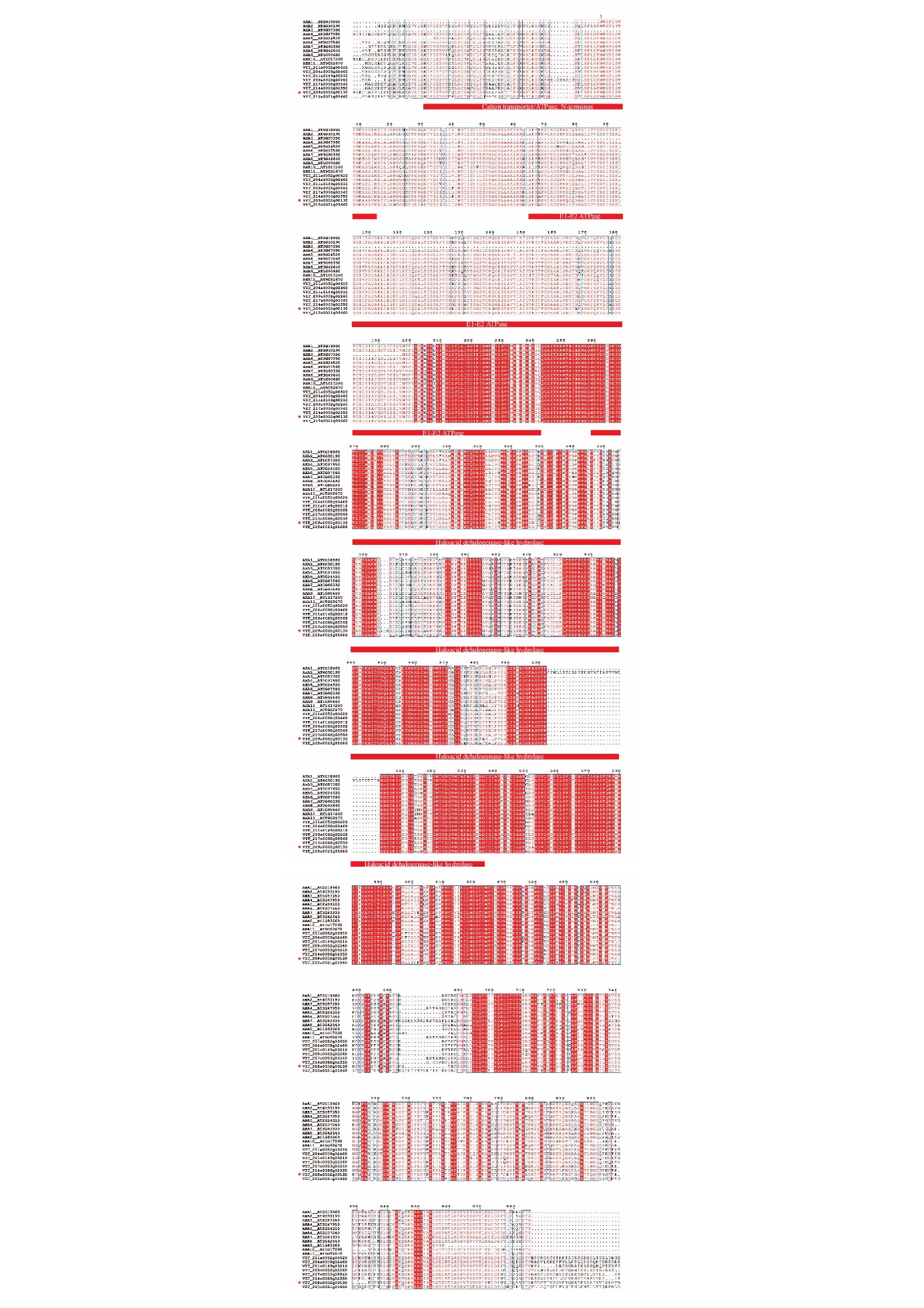

Supplement: Supplementary file 1 — Figure S1 Identification of VvERF1B‐overexpressing and mutant transgenic grape calli. Figure S2 Functional identification of VvERF1B in Arabidopsis plants. Figure S3 The experiments for detecting the binding of VvERF1B to the VvPMA10 promoter. Figure S4 CRISPR/Cas9‐mediated mutation of VvPMA10. Figure S5 Functional identification of VvPMA10 in Arabidopsis plants. Figure S6 Y1H experiments for detecting the binding of VvMYC2 to cis‐elements in the VvPMA10 promoter. Figure S7 CRISPR/Cas9‐mediated mutation of VvMYC2. Figure S8 Expression analysis of VvMYC2 in VvERF1B transgenic calli (a) and roots (b). Figure S9 Identification of transgenic grape calli with altered VvERF1B and VvMYC2 expression or mutant VvMYC2. Figure S10 Effects of the exogenous application of JA on the NaHCO3 stress tolerance of grapevines. Figure S11 Expression analysis of the JA synthesis‐related genes (a‐c), VvACS3 (d), VvERF1B (e), VvMYC2 (f), and VvPMA10 (g) in grapevine roots treated with ethylene or MeJA for 6 h. Figure S12 The phylogenetic relationships of ERF1 proteins from Arabidopsis thaliana and grapevine. Figure S13 Alignment of the deduced amino acid sequence of plasma membrane H+‐ATPases from Arabidopsis thaliana (AHA) and grapevine (PMA). [file PBI-23-1076-s002.docx]
